# Biliary-Atresia-Associated Mannosidase-1-Alpha-2 Gene Regulates Biliary and Ciliary Morphogenesis and Laterality

**DOI:** 10.3389/fphys.2020.538701

**Published:** 2020-10-30

**Authors:** Juhoon So, Mylarappa Ningappa, Joseph Glessner, Jun Min, Chethan Ashokkumar, Sarangarajan Ranganathan, Brandon W. Higgs, Dong Li, Qing Sun, Lori Schmitt, Amy C. Biery, Steven Dobrowolski, Christine Trautz, Leah Fuhrman, Molly Christine Schwartz, Nikolai Thomas Klena, Joseph Fusco, Krishna Prasadan, Morayooluwa Adenuga, Nada Mohamed, Qi Yan, Wei Chen, William Horne, Anil Dhawan, Khalid Sharif, Deirdre Kelly, Robert H Squires, George K. Gittes, Hakon Hakonarson, Victor Morell, Cecilia Lo, Shankar Subramaniam, Donghun Shin, Rakesh Sindhi

**Affiliations:** ^1^Department of Developmental Biology, McGowan Institute of Regenerative Medicine, University of Pittsburgh, Pittsburgh, PA, United States; ^2^Hillman Center for Pediatric Transplantation of the Children’s Hospital of Pittsburgh of University of Pittsburgh Medical Center (UPMC), Pittsburgh, PA, United States; ^3^Center for Applied Genomics of the Children’s Hospital of Philadelphia, Philadelphia, PA, United States; ^4^Departments of Bioengineering, Cellular and Molecular Medicine, and Computer Science and Engineering, University of California San Diego, La Jolla, CA, United States; ^5^Division of Pediatric Pathology, Department of Pathology, Children’s Hospital of Pittsburgh of UPMC, Pittsburgh, PA, United States; ^6^Histology Core Laboratory, Children’s Hospital of Pittsburgh of UPMC, Pittsburgh, PA, United States; ^7^Department of Developmental Biology, University of Pittsburgh, Pittsburgh, PA, United States; ^8^Pediatric General and Thoracic Surgery, Children’s Hospital of Pittsburgh of UPMC, Pittsburgh, PA, United States; ^9^Departments of Human Genetics and Biostatistics, Graduate School of Public Health, University of Pittsburgh, Pittsburgh, PA, United States; ^10^Richard King Mellon Foundation Institute for Pediatric Research, Children’s Hospital of Pittsburgh of UPMC, Pittsburgh, PA, United States; ^11^Paediatric Liver, GI, and Nutrition, King’s College Hospital, London, United Kingdom; ^12^Children’s Hospital of Birmingham, Birmingham, United Kingdom; ^13^Pediatric Gastroenterology, Children’s Hospital of Pittsburgh of UPMC, Pittsburgh, PA, United States; ^14^Division of Cardiac Surgery, Department of Cardiothoracic Surgery, Children’s Hospital of Pittsburgh of UPMC, Pittsburgh, PA, United States

**Keywords:** biliary atresia, liver transplantation, cilia, biliary morphogenesis, laterality

## Abstract

**Background/Aims:**

Infectious and genetic factors are invoked, respectively in isolated biliary atresia (BA), or syndromic BA, with major extrahepatic anomalies. However, isolated BA is also associated with minor extrahepatic gut and cardiovascular anomalies and multiple susceptibility genes, suggesting common origins.

**Methods:**

We investigated novel susceptibility genes with genome-wide association, targeted sequencing and tissue staining in BA requiring liver transplantation, independent of BA subtype. Candidate gene effects on morphogenesis, developmental pathways, and ciliogenesis, which regulates left-right patterning were investigated with zebrafish knockdown and mouse knockout models, mouse airway cell cultures, and liver transcriptome analysis.

**Results:**

Single nucleotide polymorphisms in Mannosidase-1-α-2 (*MAN1A2*) were significantly associated with BA and with other polymorphisms known to affect *MAN1A2* expression but were not differentially enriched in either BA subtype. In zebrafish embryos, *man1a2* knockdown caused poor biliary network formation, ciliary dysgenesis in Kupffer’s vesicle, cardiac and liver heterotaxy, and dysregulated *egfra* and other developmental genes. Suboptimal *man1a2* knockdown synergized with suboptimal EGFR signaling or suboptimal knockdown of the EGFR pathway gene, adenosine-ribosylation-factor-6, which had minimal effects individually, to reproduce biliary defects but not heterotaxy. In cultured mouse airway epithelium, *Man1a2* knockdown arrested ciliary development and motility. *Man1a2*^–/–^ mice, which experience respiratory failure, also demonstrated portal and bile ductular inflammation. Human BA liver and *Man1a2*^–/–^ liver exhibited reduced *Man1a2* expression and dysregulated ciliary genes, known to cause multisystem human laterality defects.

**Conclusion:**

BA requiring transplantation associates with sequence variants in *MAN1A2*. *man1a2* regulates laterality, in addition to hepatobiliary morphogenesis, by regulating ciliogenesis in zebrafish and mice, providing a novel developmental basis for multisystem defects in BA.

## Introduction

Biliary atresia (BA) accounts for 30–50% of all liver transplants (LTx), worldwide, and has a major public health impact which can be alleviated with a better understanding of pathogenesis (Otte et al., 1994). The heterogeneous BA phenotype includes cardiovascular or gastrointestinal anomalies in addition to atretic extrahepatic bile ducts and defies a unifying explanation (Schwarz et al., 2013). Surgical reconstruction restores bile drainage from the liver and prevents certain death during infancy, but fails over time leading to LTx in three–fourths of all children (Karrer et al., 1996). Major extrahepatic anomalies including laterality defects, which are characteristic of syndromic BA, suggest a multifactorial origin. However, minor extrahepatic anomalies in some children with isolated BA indicate that syndromic and isolated BA may also share common origins (Schwarz et al., 2013). As evidence, abnormal cilia are present on cholangiocytes in both syndromic and non-syndromic BA (Chu et al., 2012) Ciliary dysfunction predisposes to laterality defects (Afzelius, 1995). Although genetic factors are invoked in syndromic BA and remain to be proved in a controlled study, genome-wide association studies (GWAS) of cohorts predominantly comprised of isolated BA have identified multiple susceptibility genes strengthening the evidence for a common basis. The three susceptibility genes, adducin-3, glypican-1 *(GPC1)*, and adenosine-ribosylation-factor-6 *(ARF6)*, all cause biliary dysgenesis upon knockdown in zebrafish embryos likely by altering cell mobility during liver development (Cheng et al., 2013; Cui et al., 2013; Ningappa et al., 2015a,b). Additional susceptibility genes must also explain the extrahepatic anomalies of BA.

Here, we evaluate susceptibility to end-stage liver disease, which requires LTx in children with BA, independent of BA subtype. This restricts our GWAS to a cohort with homogeneous end-stage liver disease, without the heterogeneity introduced by including children without LTx, and in whom the liver disease has likely arrested at various stages. We identify *MAN1A2* as a susceptibility gene, which explains the multisystem involvement in BA. Our experimental approach combines GWAS in children with LTx for BA, zebrafish and mouse models, and liver transcriptome analysis in human BA and animal models.

## Materials and Methods

All human subjects and animal studies were performed under protocols approved by the University of Pittsburgh’s Institutional Review Board and Animal Care and Use Committee, respectively. We first used GWAS to identify SNPs associated with BA independent of BA subtype in 137 Caucasian LTx recipients at our single-center (Ningappa et al., 2015a). Targeted next-generation sequencing of the candidate, *MAN1A2*, identified potentially causal SNPs in LD with BA-associated SNPs. RT-qPCR and immunohistochemistry characterized expression of the *MAN1A2* gene and protein in affected BA liver. The effect of *MAN1A2* on biliary morphogenesis, left-right patterning, and ciliogenesis was evaluated with knockdown in zebrafish and mouse airway epithelial cell cultures, respectively (Li et al., 2015; Ningappa et al., 2015a,b). For further corroboration, we evaluated whether liver histology was abnormal in *Man1a2*^–/–^ mice, which are known to experience respiratory failure, and in mice with heterotaxy due to knockout of *Dnah11* (Bartoloni et al., 2002). *Dnah11* is representative of dysregulated ciliary genes in the liver transcriptome of human BA and *Man1a2*^–/–^ mice and *man1a2* morphant zebrafish. Details are provided in Supplementary Material.

## Results

### *MAN1A2* Is a BA Susceptibility Gene

Self-reported Caucasian BA cases and healthy controls were genotyped in three batches. The first two batches of 52 discovery cases and 1969 controls, and 53 replication cases and 400 controls, respectively, were genotyped with the HumHap Infinium 550K (Illumina) and Quad Infinium 660k (Illumina) at the Center for Applied Genomics at the Children’s Hospital of Philadelphia. Replicated candidate SNPs were assayed with TaqMan SNP Genotyping Assays in the third batch of 32 BA cases. Genotyping calls with Taqman assays were identical to those called with SNP arrays in a subset of 19 randomly selected cases from the first two batches.

A final list of 507540 and 522193 SNPs in both SNP array versions of HumHap Infinium 550K and Quad Infinium 660k were identified, respectively, after excluding missing genotype data (>0.10), Hardy-Weinberg equilibrium (HWE) test (*p* = 0.00005), and low minor allele frequency (MAF < 0.01) as described in Supplementary Material. The genotyping success rate for SNPs was 0.973456 and 0.851671 in the discovery and replication cohorts, respectively, and one BA discovery case was excluded for missing genotype data above the threshold. After multidimensional scaling analysis (MDS) to select cases and controls with similar genetic substructure, association testing was performed in 44 of 51 BA cases and 1,713 of 1,969 controls in the discovery cohort, and 45 of 53 BA cases and 347 of 400 controls in the replication cohort with the Cochran-Armitage trend test in PLINK (Supplementary Figure 1; Purcell et al., 2007). MAF were compared between cases and controls in each cohort. The candidate gene was one which contained the highest ranked two or more SNPs in the same gene (*p* < 0.005), and which retained this level of significance in the replication cohort.

In association testing, 3,322 SNPs achieved significance (*P* = 0.005) in the discovery cohort with 24 of these SNPs also remaining significant in the replication cohort (Supplementary Tables 1, 2). The top ranked flanking SNP rs6657965 in the *MAN1A2* gene demonstrated significantly higher MAF in the discovery cohort (*p* = 7.18E-05) and replication cohort (*p* = 9.73E-04) and in the 89 combined BA cases (*p* = 1.72E-08). The two top-ranked intronic SNPs rs12131109 and rs7531715 in LD (r2 > 0.8) in the *MAN1A2* gene demonstrated significantly higher MAF in the discovery cohort (*p* = 2.14E-04 and *p* = 1.89E-04, respectively), replication cohort (*p* = 9.73E-04 and *p* = 9.73E-04, respectively) and in 89 combined BA cases (4.59E-08 and *p* = 4.59E-08, respectively) (Table 1). Genome-wide, regional Manhattan plots and QQ plots illustrate these SNPs in the MAN1A2 locus (Supplementary Table 1 and Supplementary Figures 2, 3). In the combined analysis, we excluded the 32 BA cases that were genotyped with Taqman assays because beyond self-reported ethnicity, we have no data regarding where these 32 cases may fall in the MDS space. Therefore, these *p*-values are a bit less significant than *p*-values if all 121 cases and all 2060 controls with self-reported Caucasian ethnicity are compared, for example, *p* = 4.59E-08 (*n* = 89 vs. 2060) vs. *p* = 6.97E-10 (*n* = 121 vs. 2060), respectively, for rs753175. However, the odds ratio estimate remains approximately the same and SNPs achieved genome-wide significance after adjusting for False Discovery Rate using Benjamini Y method (FDR_BY) (Table 1).

**TABLE 1:** SNPs associated with BA.

SNP	Cohort	MAF	MAF	OR	*p*-values (trend test)	Adjusted *p*-value (FDR_BY)
		(BA cases) (aa/Aa/AA)	(Controls) (aa/Aa/AA)			
**rs6657965**	**Discovery**	0.2614	0.1221			–
*MAN1A2*		(*n* = 44 of 51)	(*n* = 1,713 of 1,969)	2.543	7.18E-05	
flanking_5UTR		(1/21/22)	(19/379/1,309)			
	**Replication**	0.2778	0.1383			–
chr1:		(*n* = 45 of 53)	(*n* = 347 of 400)	2.396	9.73E-04	
117694341 (hg18)		(2/21/22)	(12/72/263)			
	**Combined**	0.2697	0.1249			
		(*n* = 89 of 104)	(*n* = 2,060 of 2,369)	2.587	1.72E-08	2.83E-03
		(3/42/44)	(31/451/1,572) ***			
**rs12131109**	**Discovery**	0.2558	0.1241	2.427	2.14E-04	–
*MAN1A2*		(*n* = 44 of 51)	(*n* = 1713 of 1969)			
Intronic		(1/20/22) *	(19/387/1307)			
	**Replication**	0.2778	0.1383	2.396	9.73E-04	–
chr1:		(*n* = 45 of 53)	(*n* = 347 of 400)			
117744385(hg18)		(2/21/22)	(12/72/263)			
	**Combined**	0.2697	0.1265	2.517	4.59E-08	6.50E-03
		(*n* = 89 of 104)	(*n* = 2,060 of 2,369)			
		(3/41/44) *	(31/459/1,570)			
	**(*n* = 89) and**	0.2603	0.1265	2.497	6.97E-10	3.11E-04
	**Taqman**	(*n* = 121)	(*n* = 2060)			
	**(*n* = 32)**	(4/54/62) *	(31/459/1,570)			
**rs7531715**	**Discovery**	0.2558	0.1232	2.447	1.89E-04	–
*MAN1A2*		(*n* = 44 of 51)	(*n* = 1,713 of 1,969)			
intronic		(1/20/22) *	(19/383/1,307)			
	**Replication**	0.2778	0.1383	2.396	9.73E-04	–
chr1:		(*n* = 45 of 53)	(*n* = 347 of 400)			
117850460(hg18)		(2/21/22)	(12/72/263)			
	**Combined**	0.267	0.1257	2.533	4.59E-08	6.98E-03
		(*n* = 89 of 104)	(*n* = 2,060 of 2,369)			
		(3/41/44) *	(31/455/1,570) **			
	**(*n* = 89) and**	0.2603	0.1258	2.497	6.97E-10	3.11E-04
	**Taqman**	(*n* = 121)	(*n* = 2,060)			
	**(*n* = 32)**	(4/54/62) *	(31/455/1,570) **			

Minor allele frequencies for two intronic SNPs in the MAN1A2 gene in cases and controls selected with multidimensional scaling (MDS) plots in the discovery and replication cohorts, and in the overall population of 121 BA cases. These 121 cases include all 89 selected BA cases genotyped with SNP arrays which were used for discovery and replication, and 32 BA cases genotyped with Taqman genotyping assays. Also shown are distribution of genotypes aa, Aa, and AA in each cohort and adjusted p-values for False Discovery Rate using Benjamini Y method (FDR_BY). MAF, minor allele frequency, OR, odds ratio; BA, biliary atresia; a, minor allele; A, major allele; *, one genotype missing in BA cases; **, four genotypes missing in controls; ***, six genotypes missing in controls.

Interestingly, our BA cohort of 121 children included 98 with biliary involvement only and 23 children with extrahepatic abdominal and cardiovascular anomalies (Supplementary Table 3). The MAF of rs7531715 were 0.265 and 0.239, respectively, and not significantly different (*p* = 0.859, NS, test of proportions).

### The BA-Associated *MAN1A2* SNP Locus Encompasses Known Expressed Quantitative Trait Loci (eQTL)

**FIGURE 1:**
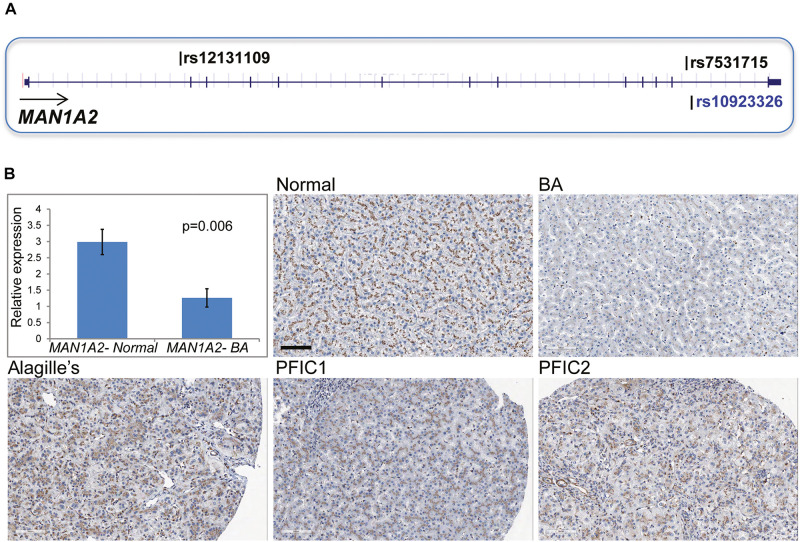
**(A)** Location of *MAN1A2* SNPs associated with BA. The SNPs rs7531715 and rs12131109 are associated with BA in GWAS. The SNP rs10923326 in LD (r2 = 1) with rs7531715 is significantly associated with gene expression in the liver. **(B)** *MAN1A2* expression in diseased BA liver. Bar diagram shows expression of the exon 6 and 7 region of *MAN1A2* in liver tissue from BA cases [male: female 1:5, mean (SEM) age at LTx, 1.33 (0.55) years] and healthy controls. The remaining five panels show diffuse strong (3+) granular immunostaining of MAN1A2 in the hepatocyte cytoplasm along pericanalicular membranes in normal human allografts, and liver from children with Alagille’s syndrome and types 1 and 2 progressive familial intrahepatic cholestasis (PFIC). Reduced MAN1A2 immunostaining is seen in BA liver (x200).

In 43 children with BA, targeted NGS of the *MAN1A2* gene inclusive of the 20 kb upstream and downstream sequences identified 498 SNP variants. Genotyping calls for rs7531715 and rs12131109 with NGS were identical to those determined with SNP arrays (*n* = 38) and Taqman assay (*n* = 2) for 40 of 43 BA cases (Supplementary Material). For three of 43 BA cases, genotyping calls made with SNP arrays and Taqman assays for the two BA-associated SNPs were identical but did not agree with genotype calls made with NGS. Nineteen SNPs in LD (*r*^2^≥ 0.8) with the two BA-associated SNPs are negatively correlated with *MAN1A2* expression at *p* ≤ 1.2067E-09 in Caucasian populations (Supplementary Table 4; Stranger et al., 2007). Among these SNPs, rs10923326 is strongly associated with *MAN1A2* expression (*p* = 3.42e-19) in the liver (Figure 1A; Schadt et al., 2008). Novel missense, nonsense, or splice-altering variants were not identified.

### Decreased *MAN1A2* Gene and Protein Expression in BA Liver

Compared with 6 normal human liver samples, liver samples from 6 children with BA demonstrated lower expression of the *MAN1A2* exon 6–7 junction (2.4-fold, *p* = 0.006, Figure 1B) and exon 10 (2.2-fold, *p* = 0.009, Supplementary Table 5) with reverse transcription quantitative PCR. MAN1A2 immunostaining revealed a diffuse cytoplasmic, peri-canalicular granular staining pattern in normal liver tissue and in diseased liver with Alagille’s syndrome and familial intrahepatic cholestasis, examples of childhood cholestatic diseases (Figure 1B). In contrast, MAN1A2 expression was markedly decreased in BA liver tissue. Staining characteristics for two normal and five BA liver samples are summarized in Supplementary Table 6.

### *man1a2* Knockdown Produces Biliary Defects in Zebrafish

**FIGURE 2:**
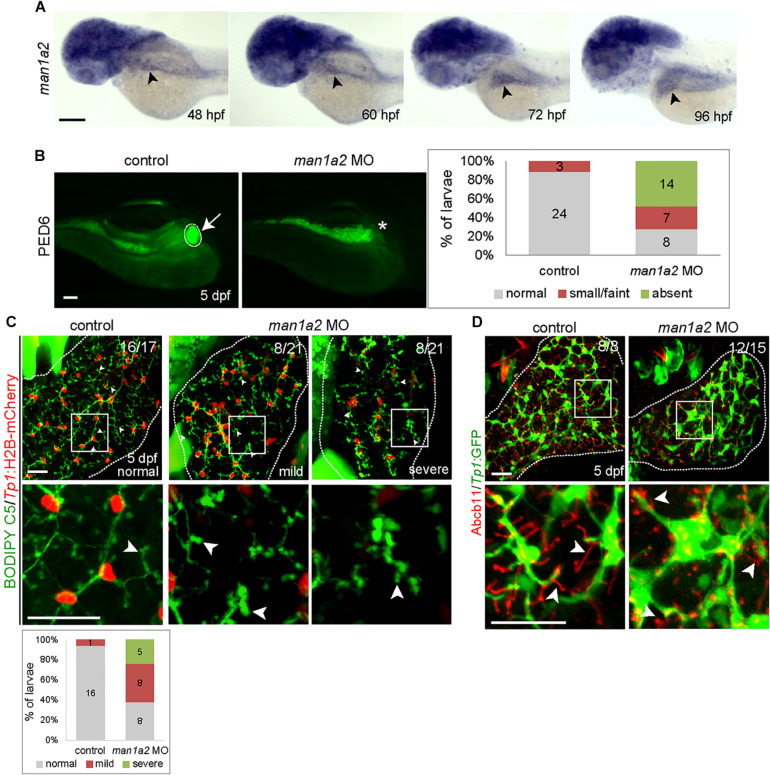
*man1a2* knockdown results in developmental biliary defects in zebrafish. **(A)** Whole-mount *in situ* hybridization image showing *man1a2* expression in developing embryos/larvae. Arrowheads point to the liver. **(B)** Epifluorescence images showing PED6 accumulation in the gallbladder. The arrow points to the gallbladder outlined by a dotted line; the asterisk denotes gallbladder location. Based on PED6 levels in the gallbladder, larvae were divided into three groups: normal, small/faint, and absent. Graph showing the percentage of larvae in each group. Numbers in the graph indicate the number of larvae in each group. **(C)** Confocal images of the liver showing the intrahepatic biliary network, as revealed by BODIPY C5 labeling (green, biliary ductal network) and *Tp1*:H2B-mCherry (red, BEC nuclei) expression. Based on the severity of biliary defects, larvae were divided into three groups: normal, mild, and severe. Higher magnification images of the square regions are also shown. Dotted lines outline the liver Graph shows the percentage of larvae in each group. Numbers in the graph indicate the number of larvae in each group. Arrows point to bile canaliculi; dotted lines outline the liver. **(D)** Confocal images of the liver showing the expression of *Tp1*:GFP (green) and Abcb11 (red) for biliary structure and hepatocyte bile canaliculi, respectively. Higher magnification images of the square regions are also shown. Arrows point to bile canaliculi; Dotted lines outline the liver. Numbers in the upper right corner are the fraction of larvae exhibiting the representative phenotype shown. Scale bars: 100 **(A,B**), 25 **(C,D)** μm.

To determine if *MAN1A2* is required for proper biliary development, we first examined and found that *man1a2 is* expressed in the liver of developing zebrafish embryos/larvae from 48 h post-fertilization (hpf) at least to 96 hpf (Figure 2A, arrowheads). To perform knockdown experiments, we designed two different MOs, ATG and splicing MOs, and validated them (Supplementary Figures 4A–C). To examine whether bile secretion from hepatocytes into biliary ducts was defective in *man1a2* morphants, we used the fluorescently labeled fatty acid reporter PED6, which is metabolized into bile in hepatocytes and accumulates in the gallbladder (Colland et al., 2004) PED6 accumulation in the gallbladder was greatly reduced in the ATG or splicing morphants compared to controls (Figure 2B and Supplementary Figure 4D). We further examined this potential biliary defect by evaluating the intrahepatic ductal network with another fluorescent lipid reporter, BODIPY C5 (Farber et al., 2001). Biliary ductules were well connected to each other in controls, frequently disconnected in *man1a2* morphants (Figure 2C), and barely connected to each other in severe cases (Figure 2C). Hepatocyte bile canaliculi were also abnormal and were very short and rounded in the morphants compared with thin and elongated canaliculi in controls (Figure 2C, arrowheads). This was further confirmed by the abnormal expression pattern of Abcb11, a bile salt pump located in bile canaliculi (Figure 2D). Moreover, BECs appeared more clustered in the morphants than in controls. Analyses of the distribution of *Tp1*:H2B-mCherry^+^ nuclei of BECs in the entire liver revealed that the percentage of BECs in cluster of four or more cells was significantly higher in the morphants than in controls (Supplementary Figure 5A). Since BECs are dispersed between 60 hpf and 5 days post-fertilization (dpf) and BEC filopodia actively protrude between 70 and 96 hpf (Ningappa et al., 2015b), this clustering phenotype observed in *man1a2* morphants suggests reduced filopodial protrusion in the morphants. In the *Tg(Tp1:GFP)* line that expresses GFP in BECs (Lorent et al., 2010; Delous et al., 2012), the length of BEC filopodia at 76 and 96 hpf and the length of interconnecting bile preductules at 5 dpf were significantly reduced in *man1a2* morphants compared to controls (Supplementary Figure 5B). Interestingly, despite the intrahepatic biliary defects, the gallbladder and the extrahepatic duct appeared morphologically normal in *man1a2* morphants (Supplementary Figure 6). Collectively, these data indicate the essential role of *man1a2* in the proper formation of hepatocyte bile canaliculi and the intrahepatic biliary network.

### *man1a2* Knockdown Results in Laterality Defects in Zebrafish

**FIGURE 3:**
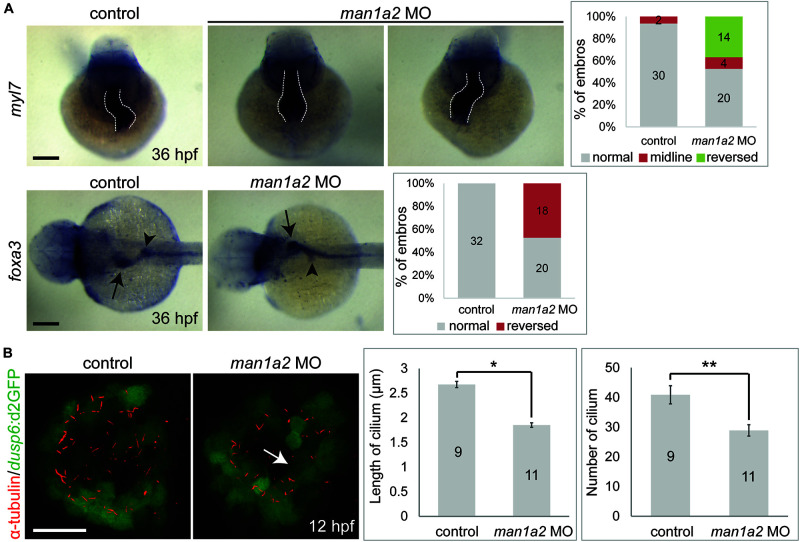
*man1a2* knockdown results in laterality defects in zebrafish. **(A)** Whole-mount *in situ* hybridization images showing *myl7* (cardiomyocytes) and *foxa3* (endodermal cells) expression at 36 hpf, which was used to determine the laterality of embryos. Three patterns of heart looping (normal, reversed, and midline) and two patterns of visceral organ position (normal and reversed) were detected and were quantified in graphs. Numbers in the graph indicate the number of larvae in each group. Arrows and arrowheads point to the liver and the dorsal pancreas, respectively; dotted lines outline the heart. Scale bars: 100 μm. **(B)** Confocal images of Kupffer’s vesicle showing the expression of *dusp6*:d2GFP (green, Kupffer’s vesicle cells) and acetylated tubulin (red, cilia). Both cilia length and number were significantly reduced in man1a2 MO-injected larvae (*n* = 11) compared to controls (*n* = 9), as shown in graphs. Scale bar: 50 μm. Error bars: ± SEM. *p < 0.001; **p < 0.005.

While analyzing biliary morphogenesis, we noticed that liver position was reversed in a significant portion of *man1a2* morphants. Since BA is associated with laterality defects (Schwarz et al., 2013), we investigated whether *man1a2* also regulates laterality. Whole-mount *in situ* hybridization with 4 different hepatic markers confirmed that liver position was reversed in 40% of *man1a2* morphants (*n* = 70, Supplementary Figure 7). Cardiac looping direction, as assessed by the expression of the cardiomyocyte marker *myl7*, was also reversed in 37% of the morphants (*n* = 38, Figure 3A). Double whole-mount *in situ* hybridization with *myl7* and *foxa3* probes revealed that the position of endoderm-derived organs, such as the liver and pancreas, was also reversed in embryos with reversed or no cardiac looping (Figure 3A). This left-right asymmetry prompted us to examine motile cilia with anti-acetylated tubulin antibody labeling in Kupffer’s vesicle in *man1a2* morphants, because cilia create a directional fluid flow which regulates left-right patterning in zebrafish (Essner et al., 2005). We used the *Tg(dusp6:d2GFP)* line to visualize Kupffer’s vesicle cells (Molina et al., 2007). Both cilia length and number were significantly reduced in the morphants at 12 hpf compared to controls indicating the essential role of *man1a2* in left-right patterning (Figure 3B).

### *man1a2* Knockdown in Zebrafish Induces Dysregulated Developmental Genes

To determine whether *man1a2* knockdown affects biliary morphogenesis via known developmental pathways, we evaluated the expression of key genes in the EGFR (*egfra, egfrb, arf6a, and arf6b*), Hedgehog (*ptch1, gli1*, and *gli2a*), and the TGF-β (*tgfb1a, tgfb1b, smad7*, and *smad9*) signaling pathways. In previous zebrafish studies, biliary dysgenesis was induced with aberrant signaling of either the EGFR or Hedgehog pathway (Cui et al., 2013; Ningappa et al., 2015b). MAN1A2 can bind to SMAD9, which can mediate TGF-β signaling (Colland et al., 2004). SMAD7 promotes cross-talk between EGFR and TGF-β signaling (Kang et al., 2012). *man1a2* knockdown induced consistent downregulation of *egfra* and upregulation of *gli1*, *smad7*, and *smad9* in all batches of zebrafish embryos that were tested (Supplementary Table 7), indicating the involvement of EGFR and TGF-β signaling in the effect of *man1a2* knockdown in the liver. RNA sequencing of pooled batches of liver tissue, one from untreated and one from morphant zebrafish revealed additional differentially expressed genes in these pathways (Table 2).

**TABLE 2:** Selected differentially expressed liver genes from human BA, *man1a2* morphant zebrafish and *Man1a2*^–/–^ mouse (see Supplementary Tables 9–12 for complete list).

Pathway	HUMAN BA Liver	*Man1a2*^–/–^ mouse liver	*man1a2* morphant zebrafish
**HEDGEHOG**	***GPC1***	***Gpc1*,** *Ptch1*	gli1*
**EGF**	***FGF23***, *FGF2, FGF7*	***Egfr, Fgf23*,** *Asap1*	egfra*
**TGF**	*TGFB2*	***Smad9*,** *Smad1, Smad3, Smad4, Tgfbr2*	smad9*, smad1, smad7*
Selected **CILIARY** genes (SYS-CILIA GOLD)	MAN1A2*, DYNC2H1, *DNAH11, IFT80, IFT81*	***Man1a2, Dync2h1*,** *Dnah2, Ift74, Invs*	*hap1*
Human **PRIMARY CILIARY DYSKINESIS** genes with laterality	*ACVR2B*, ***CENPF***, *DNAH11, GATA4*, ***HYDIN***, *OFD1, RPGR*	*Bcl9l, Ccno*, ***Cenpf***, *Cluap1, Dnaaf2, Drc1*, ***Hydin*,** *Invs, Rpgr*,	
**CONGENITAL HEART DISEASE** heterotaxy genes with extracardiac effects	***EHMT1***, *MID1*, ***RUNX1***, *GATA4*	*Ankrd11, Camta1, Crebbp, Crk*, ***Ehmt1***, *Flna, Gata6, Gja5, Gtf2Ird1, Hras, Nek2, Notch1, Nrp1, Nsd1, Pde1a, Rock2*, ***Runx1***, *Sox7, Tgfbr2*	

**These genes participate in developmental hedgehog, EGF, TGF and ciliogenesis pathways, or associate with PCD or congenital heart disease with laterality or extracardiac defects. Bold text identifies dysregulated genes in two or more species. Liver transcriptome was characterized with RNA sequencing. (^∗^) Genes characterized with RTqPCR.**

### Genetic Interaction of *man1a2*, *arf6*, and EGFR Signaling for Proper Intrahepatic Biliary Morphogenesis

**FIGURE 4:**
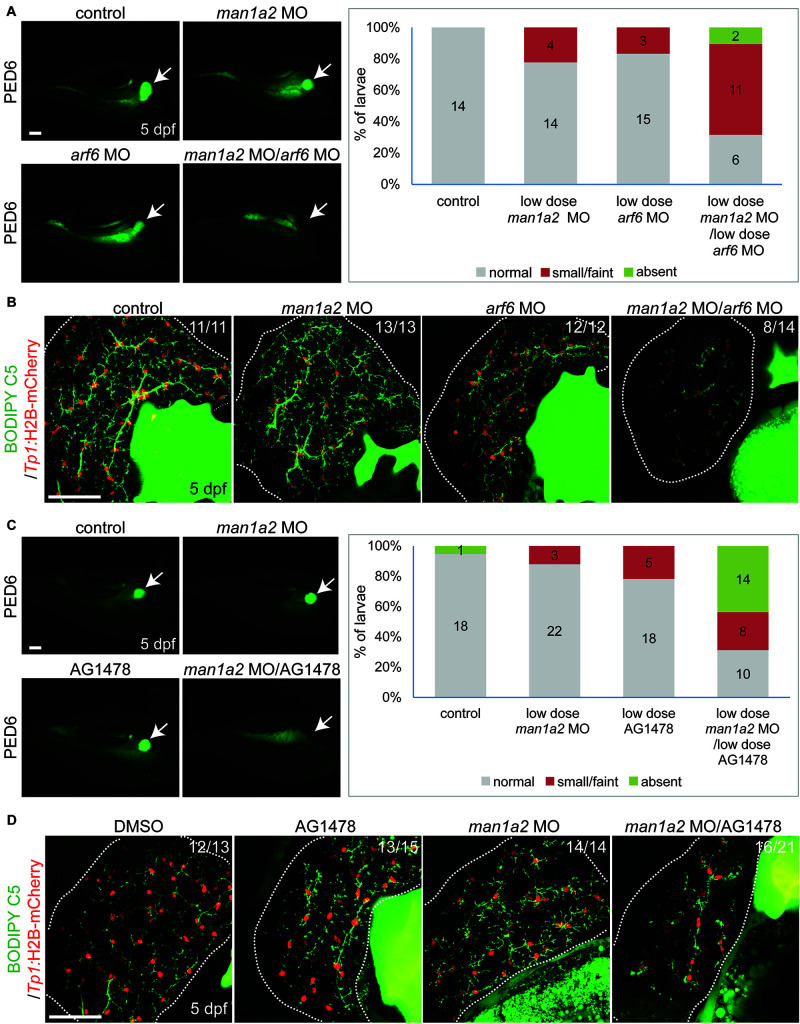
Genetic interaction among *man1a2*, *arf6* and EGFR signaling in intrahepatic biliary network formation**. (A,C)** Epifluorescence images of larvae treated with PED6 and its quantification. Suboptimal doses of *man1a2* (1.5 ng) and *arf6* (0.5 ng) MOs were singly or in combination injected into embryos at the one-cell stage **(A)**. Embryos were first injected with the suboptimal dose of *man1a2* MO (1.5 ng) and from 60 hpf treated with a suboptimal dose of AG1478 (1 μM) **(C)**. Arrows point to the gallbladder. Graphs show the percentage of larvae in each group. Numbers in the graph indicate the number of larvae in each group. **(B,D)** Confocal images of the liver showing the intrahepatic biliary network, as revealed by BODIPY-FL C5 feeding (green, biliary ductal network) and *Tp1*:H2B-mCherry (red, BEC nuclei) expression. Single injection of suboptimal doses of either *man1a2* or *arf6* MO did not impair proper intrahepatic biliary network formation, whereas their co-injection resulted in defective biliary network formation **(B)**. The biliary network formation was not impaired in larvae only treated with AG1478, whereas it was impaired in AG1478-treated larvae with *man1a2* partially knockdown **(D)**. Numbers in the upper right corner are the fraction of larvae exhibiting the representative phenotype shown. Dotted lines outline the liver. Scale bars: 50 μm.

We hypothesized that *man1a2* can genetically interact with other known genes or pathways implicated in BA (Ningappa et al., 2015a), and because *man1a2* knockdown affects EGFR and TGF-β signaling (Supplementary Table 7). We first tested if *man1a2* and *arf6* can synergistically influence proper intrahepatic biliary network formation by co-injecting suboptimal doses of *man1a2* and *arf6* MOs, which alone did not cause any obvious biliary defects (1.5 and 0.5 ng, respectively). Compared to single-injected larvae, co-injected larvae demonstrated greatly reduced PED6 accumulation in the gallbladder (Figure 4A) and a severely impaired intrahepatic ductal network in 57% of co-injected larvae on BODIPY C5 labeling (*n* = 14; Figure 4B). Intriguingly, liver position was not reversed in the co-injected larvae (data not shown). Next, we tested if *man1a2* and EGFR signaling can synergistically influence biliary morphogenesis. Previously, blocking EGFR signaling in zebrafish larvae with the EGFR inhibitor AG1478 resulted in biliary morphogenesis defects (Ningappa et al., 2015b). Zebrafish embryos were first injected with the suboptimal dose of *man1a2* MO and then treated from 60 hpf with a suboptimal dose of AG1478 (1 μM). This treatment impaired proper biliary network formation, as accessed by PED6 (Figure 4C) and BODIPY C5 (Figure 4D) labeling. Altogether, these data suggest that *man1a2* can regulate intrahepatic biliary network formation via interactions with *arf6*, another BA susceptibility gene, and EGFR signaling.

### *Man1a2* Knockdown Impairs Motile Cilia Function in Mouse Tracheal Cells

**FIGURE 5:**
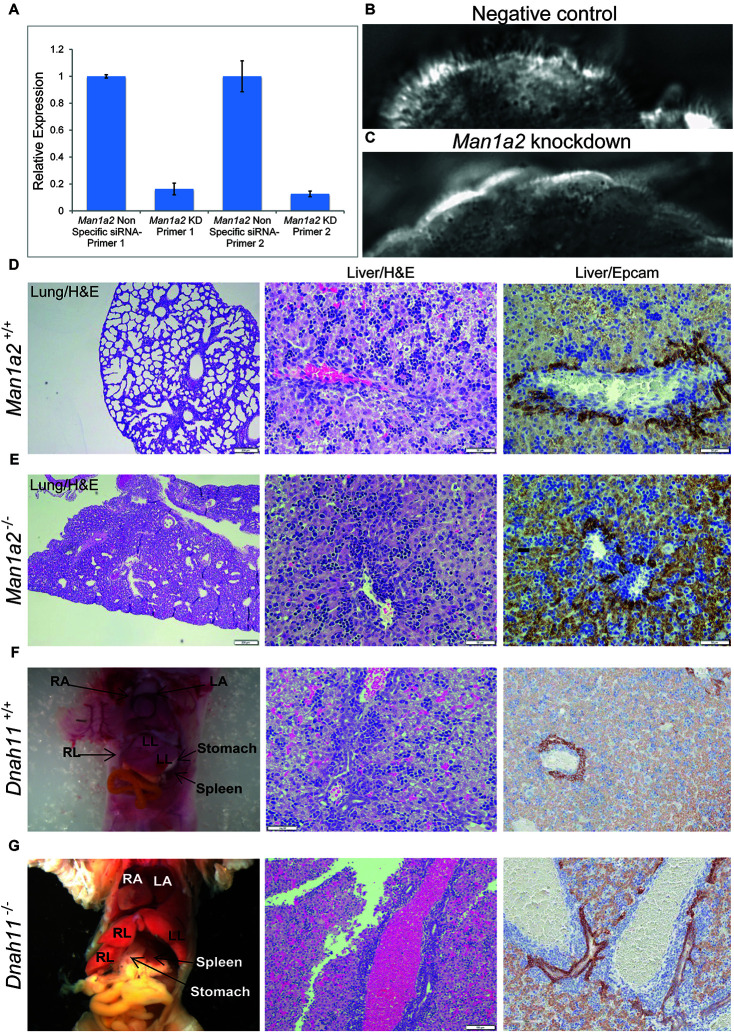
*Man1a2* knockdown produces cilia defects in reciliating mouse respiratory epithelia. **(A)** Relative expression of *Man1a2* in respiratory epithelia after siRNA transfection. **(B)** Control epithelia are well ciliated with well-coordinated ciliary beat with a full stroke (Supplementary Video 1). **(C)** *Man1a2*-siRNA-transfected respiratory epithelia exhibited very short cilia that were mostly immotile except for a few areas with slow dyskinetic ciliary beat with incomplete stroke (Supplementary Video 2). **(D,E)** *Man1a2* knockout mice with lung involvement also exhibit liver inflammation. **(D)** *Man1a2*^+/+^ liver shows a portal area with indistinct bile ducts, abundant extramedullary hematopoiesis (EMH, H&E x200), and minimal ductular reaction (Epcam x200). The lung shows expanded alveoli and thin interalveolar septa. **(E)** *Man1a2*^–/–^ liver shows expanded portal areas with inflammation including EMH (H&E) and ductular reaction (Epcam x 200). The lung shows thick septa with sparse, unexpanded alveoli. The *Man1a2*^–/–^ liver transcriptome shows several dysregulated ciliary genes, including *Dnah11* which causes PCD (Table 2 and Supplementary Table 10). **(F,G)**. *Dnah11*^–/–^ mice with situs inversus also exhibit biliary inflammation. **(F)** *Dnah11*^+/+^ mouse with normal location of heart and abdominal organs which includes two left and one right liver lobes, normal liver histology with EMH and some bile ducts in portal area. **(G)** *Dnah11*^–/–^ null mouse shows situs inversus with cardiac heterotaxy, inverted lobation of the liver (two right lobes), right-sided stomach and more centrally located spleen. Liver shows portal congestion and bile duct proliferation. All experiments were performed in triplicate.

Because specification of left-right patterning requires motile cilia function in the embryonic node in mice or Kupffer’s vesicle in zebrafish embryos, we evaluated *Man1a2* knockdown effects in reciliating mouse respiratory epithelia as a proxy for the embryonic node. In our previous studies, genes required for motile cilia function in the trachea are also usually required for motile cilia function for left-right patterning in the embryonic node (Li et al., 2015). Many such genes are known to cause human primary ciliary dyskinesia (PCD) (Bergström et al., 2012). Transfection of *Man1a2* siRNA in the respiratory epithelia before placing cells into suspension culture for reciliation showed more than 80% reduction of *Man1a2* expression by qPCR (Figure 5A), and marked inhibition of ciliogenesis resulting in very short cilia and markedly reduced percent ciliation in the *Man1a2* siRNA treated cultures (Figure 5C). Videomicroscopy showed that most of these cilia were immotile, with only a few areas exhibiting slow dyskinetic ciliary motion in transfected cells (Supplementary Video 2). In comparison, scrambled siRNA knockdown had no effect on ciliogenesis and the ciliary motion remained robust with normal pattern of ciliary beat (Figure 5B and Supplementary Video 1). Together these findings indicate an important role for *Man1a2* in regulating motile cilia function.

### *Man1a2*^–/–^ Mice Exhibit Bile Duct Inflammation and Dysregulated *Man1a2* Exons

To corroborate the effects of *Man1a2* on liver development, we bred *Man1a2^+/–^* mice with heterozygous exon 2 deletion (Tremblay et al., 2007). We confirmed that compared with *Man1a2^+/+^* WT mice, *Man1a2*^–/–^ null mice exhibit unexpanded alveoli, thick interalveolar lung septa, and respiratory failure, and a grossly normal liver as reported previously. However, upon detailed histological evaluation, the *Man1a2*^–/–^ liver showed portal expansion, inflammation and ductular reaction, suggestive of biliary inflammation, compared with *Man1a2^+/+^* WT liver (Figure 5). The reverse transcription quantitative PCR analysis showed complete loss of exon 2 expression in the lung and the liver. Reduced expression of mid-level (exon 5–6 junction) and terminal (exon 11–12 junction) exons in both organs in null mice, compared with WT achieved significance in the lung, and not the liver (Supplementary Table 8).

### *Man1a2* Knockdown Leads to Decreased Cilia Development in Mouse Lung

**FIGURE 6:**
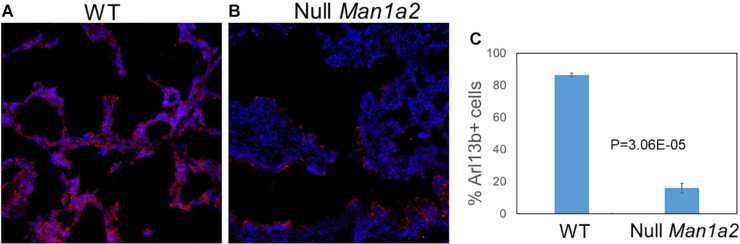
*Man1a2* knockdown in mouse leads to decreased cilia development in lung. Confocal microscopy showed the cilia marker Arl13b (red) and nuclei (blue, Dapi) in the lung from the **(A)** WT (*Man1a2*^+/+^) and **(B)** Null *Man1a2*^(−/−)^ pups. Scale bars: 50 μm. **(C)** The bar diagram showed the percent Arl13b+ cells (% Dapi) in lung from four WT and four Null *Man1a2* pups. Error bars: ± SEM.

To evaluate cilia formation, immunostaining of the ciliary marker Arl13b was performed in explanted lung and liver tissue from WT and null pups. Explanted lung tissue from Null *Man1a2* pups demonstrated 15.96% Arl13b+ cells compared with 86.5% Arl13b+ cells in WT *Man1a2* pups (*P* = 3.05E-05) (Figure 6). Arl13b staining protocols showed inconsistent staining in explanted liver tissue.

### *Man1a2* Regulates Ciliogenesis

Given dysregulated developmental pathways in *man1a2* morphant zebrafish liver, we asked whether developmental and cilia pathways, and genes associated with aberrant laterality in humans were also dysregulated in the whole transcriptome of *Man1a2*^–/–^ liver and lung and human BA liver (De Luca et al., 2010; Shiraishi and Ichikawa, 2012; Van Dam et al., 2013; Andersen et al., 2014; Bergström et al., 2012; Cerami et al., 2011). Reverse transcription qPCR showed reduced MAN1A2 expression (Supplementary Tables 5, 8) and RNA sequencing showed dysregulated genes in EGF, TGF, hedgehog and ciliogenesis pathways in human BA and *Man1a2*^–/–^ mouse liver and lung compared with corresponding controls (Table 2 and Supplementary Tables 9–11). Additionally, of 101 genes reportedly associated with PCD or human congenital heart disease with extracardiac and laterality defects, dysregulation of 7, 29, and 25 genes, respectively, was seen in human BA liver, *Man1a2*^–/–^ liver and *Man1a2*^–/–^ lung (Supplementary Tables 9–11; De Luca et al., 2010; Shiraishi and Ichikawa, 2012; Andersen et al., 2014; Bergström et al., 2012). In the BA liver, dysregulated *DNAH11* is illustrative of ciliary genes which are implicated in multisystem laterality defects in PCD and heterotaxy (Bartoloni et al., 2002). Therefore, we asked whether previously reported situs inversus in the *Dnah11*^–/–^ null mouse was accompanied by biliary pathology. Detailed histologic evaluation revealed bile ductular proliferation consistent with bile duct inflammation which is also seen in BA in *Dnah11*^–/–^ mice, compared with a *Dnah11^+/+^* WT mouse (Figure 5). Therefore, a dysregulated *MAN1A2* gene can contribute to multisystem developmental defects directly, via differential expression of exon-level transcripts in different tissues, or via genes which regulate ciliogenesis and left-right patterning.

## Discussion

Conducted in an exploratory cohort of 137 Caucasian children with BA, all of whom received LTx at our center, GWAS and biological interrogation of the *MAN1A2* locus in zebrafish and mouse models implicates ciliary dysgenesis as a common potential contributor to the syndromic and non-syndromic subtypes of BA. Three SNPs in this gene locus, two intronic and the third in the 5′prime region emerged as the top ranked SNP trio in screening and replication cohorts, each tested with a different version of the SNP array. In the combined cohort, these SNPs achieved genome-wide significance with OR exceeding 2 and *p* < 10E-08, statistical attributes which have characterized previous GWAS conducted in modest cohorts. Because of these results, the association of *MAN1A2* with BA can at first be dismissed as one of many, for example, *GPC1*, *ADD3, EFEMP1*, and *ARF6*, which have failed exact replication in multiple studies. However, the ADD3 locus has been replicated on the basis of SNPs that are intronic in Chinese and 3′ regulatory region in Caucasian children. Thus, the metric for replication may have to evolve to include gene function and its associated mechanism or pathway, and biological validation. Remarkably, each of these five potential susceptibility genes are members of the ciliogenesis and planar polarity effector network of proteins, or CPLANE network. The CPLANE proteins interact with the ciliopathy-associated protein Jbts17 at the base of cilia to recruit intraflagellar transport (IFT-A) proteins. Sequence variants in genes for CPLANE proteins induce ciliopathies in mice and humans.

This association between *MAN1A2* and BA is applicable to Caucasians but not East Asians, in whom the SNP locus is monomorphic. Further, although the associated SNPs do not affect *MAN1A2* expression in public databases, both are in LD (r2 > 0.8) with several others identified by targeted sequencing of subject samples, which are known *MAN1A2* eQTLs (Supplementary Table 4; Stranger et al., 2007). Of these SNPs, rs10923326 is strongly associated with *MAN1A2* gene expression (*p* = 3.42E-19) in previous human liver studies (Schadt et al., 2008). This eQTL function explains decreased expression of *MAN1A2* in the BA liver (2.4-fold, *p* = 0.006) (Figure 1). This translates into loss of peri-canalicular distribution of the MAN1A2 protein in BA compared with normal liver and liver with other childhood cholestatic diseases (Figure 1B). These findings have functional relevance because *man1a2* knockdown impairs intrahepatic biliary network formation and bile drainage from the liver in zebrafish larvae (Figures 2, 3A). The accompanying heterotaxy of the liver, the pancreas and the heart is a unique effect of the *man1a2* gene, which has not been reported for any BA susceptibility gene.

This novel effect of *man1a2* knockdown, i.e., regulating left-right patterning in addition to biliary morphogenesis is independent of its effect on developmental pathways such as EGF (Supplementary Table 7; Molina et al., 2007). We evaluated whether the EGFR pathway, implicated in BA in our previous work is a representative downstream target of *man1a2* knockdown. We find that suboptimal *man1a2* knockdown synergizes with suboptimal MO-mediated knockdown of *arf6*, or suboptimal AG1478-mediated inhibition of EGFR signaling to produce biliary dysgenesis and inhibit bile drainage in zebrafish larvae (Figure 5). Therefore, *man1a2* dysregulation alters EGFR signaling or can work synergistically with other BA susceptibility genes such as *arf6*, to limit bile duct development in BA. Because neither experiment combining suboptimal knockdown produced heterotaxy, *man1a2* is the sole contributor to heterotaxy via its effect on cilia, which regulates normal left-right patterning during development (Afzelius, 1995). *man1a2* knockdown reduces the length and numbers of cilia in Kupffer’s vesicles of zebrafish (Figure 3B). Further, siRNA-mediated *Man1a2* knockdown arrests ciliary development and motility in mouse airway epithelia (Figure 5C and Supplementary Videos 1, 2) and in line with the decreased staining of the ciliary protein Arl13b in lungs of Null Man1a2 mice pups (Figure 6).

Corroborative evidence comes from detailed evaluation of the liver from previously described mouse models with known cardiothoracic defects. Null mice with *Man1a2*^–/–^ exon 2 deletion which are known to die of respiratory failure due to poorly expanded alveoli also show portal expansion, inflammation and ductular reaction, suggestive of biliary inflammation in our studies (Figure 5E; Bergström et al., 2012). Despite loss of exon 2 expression in both organs, the liver phenotype is not as pronounced as would be expected from the lethal pulmonary lesions, partly because the accompanying reduced expression of mid-level and terminal *Man1a2* exons is less pronounced in the liver than the lung in null mice. *MAN1A2* is highly prone to differential splicing (Al-Balool et al., 2011). A unifying feature is that differentially expressed genes in the *Man1a2*^–/–^ liver and lung as well as liver from *man1a2* morphant zebrafish and the human BA liver participate in EGF, TGF and hedgehog signaling pathways (Table 2 and Supplementary Tables 9–12). The human BA and *Man1a2*^–/–^ transcriptomes also demonstrate dysregulated ciliary genes, many of which cause human PCD, or congenital heart disease with laterality and/or extracardiac defects (De Luca et al., 2010; Shiraishi and Ichikawa, 2012; Andersen et al., 2014; Bergström et al., 2012). The final evidence includes features of bile duct inflammation in the liver from *DNAH11*^–/–^ mice, which are known to exhibit situs inversus (Bartoloni et al., 2002). *Dnah11* is differentially expressed along with other ciliary genes in the human BA liver.

Other mechanisms by which *MAN1A2* may contribute to BA include the role of the MAN1A2 enzyme in removing excess mannose residues during maturation of glycoproteins (Tremblay and Herscovics, 2000). In experimental models, reduced MAN1A2 enzyme activity is associated with uncleaved mannose residues in EGF receptors, which resist phosphorylation and bind poorly to downstream targets (Kawashima et al., 2009). This effect can limit EGFR-dependent branching morphogenesis, which is essential for the proper development of epithelial tubular networks such as the biliary tree (Cabernard and Affolter, 2005). On the endothelial cell surface, glycoproteins with high mannose content promote inflammation via enhanced leukocyte adhesion (Chacko et al., 2011). Loss of extrahepatic bile ducts in children with BA is associated with a fibroinflammatory process (Schwarz et al., 2013).

A provocative inference from zebrafish experiments is that multisystem biliary network and laterality defects can arise from dysregulation of single gene such as *MAN1A2*, dysregulation of multiple genes with each contributing small effects, such as *ARF6* and *MAN1A2*, or via downstream effects cascading through several interacting pathways, e.g., the EGF and perhaps the hedgehog and the TGF-β pathways. The implications for the heterogeneous BA phenotype and multiple associated susceptibility genes are that non-syndromic and syndromic BA also represent a phenotypic continuum arising from common mechanisms mediated by multiple genes, for example, ciliary dysgenesis, which affects human cholangiocytes from both disease variants (Afzelius, 1995). The lack of differential enrichment of rs7531715 in BA with or without extrahepatic anomalies supports this contention.

To evaluate the role of the CPLANE network and the *MAN1A2 gene*, we have recently initiated a large scale GWAS with 756 Caucasian BA patients and 4k plus matched controls. A targeted analysis reveals that the *MAN1A2* SNP rs12131109 achieves a *p*-value of 0.05417. This is not surprising because most associations identified in large cohorts have odds ratios in the 1.1–1.2 range. Our ongoing investigations represent the largest GWAS study ever initiated for this disease using a 10-year collection from the US-based CHiLDReN network and our center. The strength of our hypothesis that ciliary dysgenesis is a common basis of BA resides in the fact that each previously reported susceptibility gene from three different groups worldwide, independently points to the CPLANE network, achieving a replication that cannot be dismissed as coincidental.

## Data Availability Statement

The datasets presented in this study can be found in online repositories. The names of the repository/repositories and accession number(s) can be found below: https://www.ncbi.nlm.nih.gov/geo/, GSE138251, GSE138399, and GSE159720, embargoed for 6 months.

## Ethics Statement

The studies involving human participants were reviewed and approved by the Institutional Review Board, University of Pittsburgh. Written informed consent to participate in this study was provided by the participants’ legal guardian/next of kin. The animal study was reviewed and approved by Animal Care and Use Committee, University of Pittsburgh.

## Author Contributions

JS and MN conducted, described zebrafish, and human genomic studies, respectively, AB, SD, and BH conducted, supervised targeted NGS. JG, JM, DL, QS, QY, and BH designed, implemented, cross-checked genotyping, and sequencing analyses. LS and SR analyzed immunohistochemistry. MS, NK, and CL designed, conducted, described mouse airway cell culture, and Dnah11^–/–^ mouse experiments. JF, KP, GG, MA, and NM conducted, described Man1a2^–/–^ mouse studies. AB, CA, CT, and LF recruited subjects, coordinated study, prepared genomic material. AD, RHS, VM, HH, SS, KS, DK, CL, and WC interpreted findings for respective human therapeutic areas, edited manuscript. SS supervised the bioinformatics and systems biology analysis. DS designed, described, supervised zebrafish experiments. RS designed, supervised the entire project and wrote the manuscript. All authors contributed to the article and approved the submitted version.

## Conflict of Interest

The authors declare that the research was conducted in the absence of any commercial or financial relationships that could be construed as a potential conflict of interest.

## Abbreviations

ARF6: adenosine-ribosylation-factor-6
BA: biliary atresia
GPC1: glypican-1
GWAS: genome-wide association study
LD: linkage disequilibrium
LTx: Liver transplantation
MAN1A2: Mannosidase-1- α -2
NGS: next-generation sequencing
PCD: Primary ciliary dyskinesia
RTqPCR: Reverse transcription quantitative polymerase chain reaction
SNP: single nucleotide polymorphisms.

## References

[B1] Afzelius B. A. (1995). Situs inversus and ciliary abnormalities. What is the connection? *Int. J. Dev. Biol.* 39 839–844. 8645568

[B2] Al-Balool H. H. Weber D. Liu Y. Wade M. Guleria K. Nam P. L. P. (2011). Post-transcriptional exon shuffling events in humans can be evolutionarily conserved and abundant. *Genome Res.* 21 1788–1799. 10.1101/gr.116442.110 21948523 PMC3205564

[B3] Andersen T. A. Troelsen Kde L. Larsen L. A. (2014). Of mice and men: molecular genetics of congenital heart disease. *Cell Mol. Life Sci.* 71 1327–1352. 23934094 10.1007/s00018-013-1430-1 PMC3958813

[B4] Bartoloni L. Blouin J. L. Pan Y. Gehrig C. Maiti A. K. Scamuffa N. (2002). Mutations in the DNAH11 (axonemal heavy chain dynein type 11) gene cause one form of situs inversus totalis and most likely primary ciliary dyskinesia. *Proc. Natl. Acad. Sci. U.S.A.* 99 10282–10286. 10.1073/pnas.152337699 12142464 PMC124905

[B5] Bergström S.-E. King T. E. Jr. Hollingsworth H. (2012). *Primary Ciliary Dyskinesia (Immotile-Cilia Syndrome). UpToDate.com.* Available online at: http://www.uptodate.com/contents/primary-ciliary-dyskinesia-immotile-cilia-syndrome (accessed September 8, 2016).

[B6] Cabernard C. Affolter M. (2005). Distinct roles for two receptor tyrosine kinases in epithelial branching morphogenesis in *Drosophila*. *Dev. Cell* 9 831–842. 10.1016/j.devcel.2005.10.008 16326394

[B7] Cerami E. G. Gross B. E. Demir E. Rodchenkov I. Babur O. Anwar N. (2011). Pathway commons, a web resource for biological pathway data. *Nucleic Acids Res.* 2011 D685–D690. 10.1093/nar/gkq1039 PMC3013659 21071392

[B8] Chacko B. K. Scott D. W. Chandler R. T. Patel R. P. (2011). Endothelial surface N-glycans mediate monocyte adhesion and are targets for anti-inflammatory effects of peroxisome proliferator-activated receptor γ ligands. *J. Biol. Chem.* 286 38738–38747. 10.1074/jbc.m111.247981 21911496 PMC3207389

[B9] Cheng G. Tang C. S. Wong E. H. Cheng W. W. So M. T. Miao X. (2013). Common genetic variants regulating ADD3 gene expression alter biliary atresia risk. *J. Hepatol.* 59 1285–1291. 10.1016/j.jhep.2013.07.021 23872602

[B10] Chu A. S. Russo P. A. Wells R. G. (2012). Cholangiocyte cilia are abnormal in syndromic and non- syndromic biliary atresia. *Mod. Pathol.* 25 751–757. 10.1038/modpathol.2011.212 22301700 PMC3341539

[B11] Colland F. Jacq X. Trouplin V. Mougin C. Groizeleau C. Hamburger A. (2004). Functional proteomics mapping of a human signaling pathway. *Genome Res.* 14 1324–1332. 10.1101/gr.2334104 15231748 PMC442148

[B12] Cui S. Leyva-Vega M. Tsai E. A. EauClaire S. F. Glessner J. T. Hakonarson H. (2013). Evidence from human and zebrafish that GPC1 is a biliary atresia susceptibility gene. *Gastroenterology* 144 1107–1115. 10.1053/j.gastro.2013.01.022 23336978 PMC3736559

[B13] De Luca A. Sarkozy A. Consoli F. Ferese R. Guida V. Dentici M. L. (2010). Familial transposition of the great arteries caused by multiple mutations in laterality genes. *Heart* 96 673–677. 10.1136/hrt.2009.181685 19933292

[B14] Delous M. Yin C. Shin D. Ninov N. Carten J. D. Pan L. (2012). Sox9b is a key regulator of pancreaticobiliary ductal system development. *PLoS Genet.* 8:e1002754 10.1371/journal.pone.1002754 PMC3375260 22719264

[B15] Essner J. J. Amack J. D. Nyholm M. K. Harris E. B. Yost J. (2005). Kupffer’s vesicle is a ciliated organ of asymmetry in the zebrafish embryo that initiates left-right development of the brain, heart and gut. *Development* 132 1247–1260. 10.1242/dev.01663 15716348

[B16] Farber S. A. Pack M. Ho S. Y. Johnson I. D. Wagner D. S. Dosch R. (2001). Genetic analysis of digestive physiology using fluorescent phospholipid reporters. *Science* 292 1385–1388. 10.1126/science.1060418 11359013

[B17] Kang M. Choi S. Jeong S. J. Lee S. A. Kwak T. K. Kim H. (2012). Cross-talk between TGFβ1 and EGFR signalling pathways induces TM4SF5 expression and epithelial-mesenchymal transition. *Biochem. J.* 443 691–700. 10.1042/bj20111584 22292774

[B18] Karrer F. M. Price M. R. Bensard D. D. Sokol R. J. Narkewicz M. R. Smith D. J. (1996). Long-term results with the Kasai operation for biliary atresia. *Arch. Surg.* 131 493–496. 10.1001/archsurg.1996.01430170039006 8624194

[B19] Kawashima N. Yoon S. J. Itoh K. Nakayama K. (2009). Tyrosine kinase activity of epidermal growth factor receptor is regulated by GM3 binding through carbohydrate to carbohydrate interactions. *J. Biol. Chem.* 284 6147–6155. 10.1074/jbc.m808171200 19124464

[B20] Li Y. Klena N. Gabriel G. Liu X. Kim A. J. Lemke K. (2015). Global genetic analysis in mice unveils central role for cilia in congenital heart disease. *Nature* 521 520–524. 10.1038/nature14269 25807483 PMC4617540

[B21] Lorent K. Moore J. C. Siekmann A. F. Lawson N. Pack M. (2010). Reiterative use of the notch signal during zebrafish intrahepatic biliary development. *Dev. Dyn.* 239 855–864. 10.1002/dvdy.22220 20108354 PMC4677821

[B22] Molina G. A. Watkins S. C. Tsang M. (2007). Generation of FGF reporter transgenic zebrafish and their utility in chemical screens. *BMC Dev. Biol.* 7:62. 10.1186/1471-213X-7-62 17553162 PMC1904198

[B23] Ningappa M. Min J. Higgs B. W. Ashokkumar C. Ranganathan S. Sindhi R. (2015a). Genome-wide association (studies) in biliary atresia. *Wiley Interdiscip. Rev. Syst. Biol. Med.* 7 267–273. 10.1002/wsbm.1303 25963027

[B24] Ningappa M. So J. Glessner J. Ashokkumar C. Ranganathan S. Min J. (2015b). The role of ARF6 in biliary atresia. *PLoS One* 10:e0138381. 10.1371/journal.pone.0138381 26379158 PMC4574480

[B25] Otte J. B. de Ville, de Goyet J. Reding R. Hausleithner V. Sokal E. (1994). Sequential treatment of biliary atresia with Kasai portoenterostomy and liver transplantation: a review. *Hepatology* 20(1 Pt 2), 41S–48S. 8005579 10.1016/0270-9139(94)90272-0

[B26] Purcell S. Neale B. Todd-Brown K. Thomas L. Ferreira M. A. R. Bender D. (2007). PLINK: a tool set for whole-genome association and population-based linkage analyses. *Am. J. Hum. Genet.* 81 559–575. 10.1086/519795 17701901 PMC1950838

[B27] Schadt E. E. Molony C. Chudin E. Hao K. Yang X. Lum P. Y. (2008). Mapping the genetic architecture of gene expression in human liver. *PLoS Biol.* 6:e107. 10.1371/journal.pbio.0060107 18462017 PMC2365981

[B28] Schwarz K. B. Haber B. H. Rosenthal P. Mack C. L. Moore J. Bove K. (2013). Extrahepatic anomalies in infants with biliary atresia: results of a large prospective North American multicenter study. *Hepatology* 58 1724–1731. 10.1002/hep.26512 23703680 PMC3844083

[B29] Shiraishi I. Ichikawa H. (2012). Human heterotaxy syndrome - from molecular genetics to clinical features, management, and prognosis. *Circ. J.* 76 2066–2075. 22864291 10.1253/circj.cj-12-0957

[B30] Stranger B. E. Nica A. C. Forrest M. S. Dimas A. Bird C. P. Beazley C. (2007). Population genomics of human gene expression. *Nat. Genet.* 39 1217–1224. 17873874 10.1038/ng2142 PMC2683249

[B31] Tremblay L. O. Herscovics A. (2000). Characterization of a cDNA encoding a novel human Golgi alpha 1, 2-mannosidase (IC) involved in N-glycan biosynthesis. *J. Biol. Chem.* 275 31655–31660. 10.1074/jbc.m004935200 10915796

[B32] Tremblay L. O. Nagy Kovács E. Daniels E. (2007). Respiratory distress and neonatal lethality in mice lacking Golgi alpha1,2-mannosidase IB involved in N-glycan maturation. *J. Biol. Chem.* 82 2558–2566. 10.1074/jbc.m608661200 17121831

[B33] Van Dam T. J. Wheway G. Slaats G. G. (2013). SYSCILIA Study Group, Huynen MA, Giles RH. The SYSCILIA gold standard (SCGSv1) of known ciliary components and its applications within a systems biology consortium. *Cilia* 2:7. 10.1186/2046-2530-2-7 PMC3674929 23725226

