# Supplementary figures and images for: Biliary-Atresia-Associated Mannosidase-1-Alpha-2 Gene Regulates Biliary and Ciliary Morphogenesis and Laterality
Source: Front Physiol. 2020 Oct 30;11:538701. doi: 10.3389/fphys.2020.538701 (PMC7662016; doi:10.3389/fphys.2020.538701)

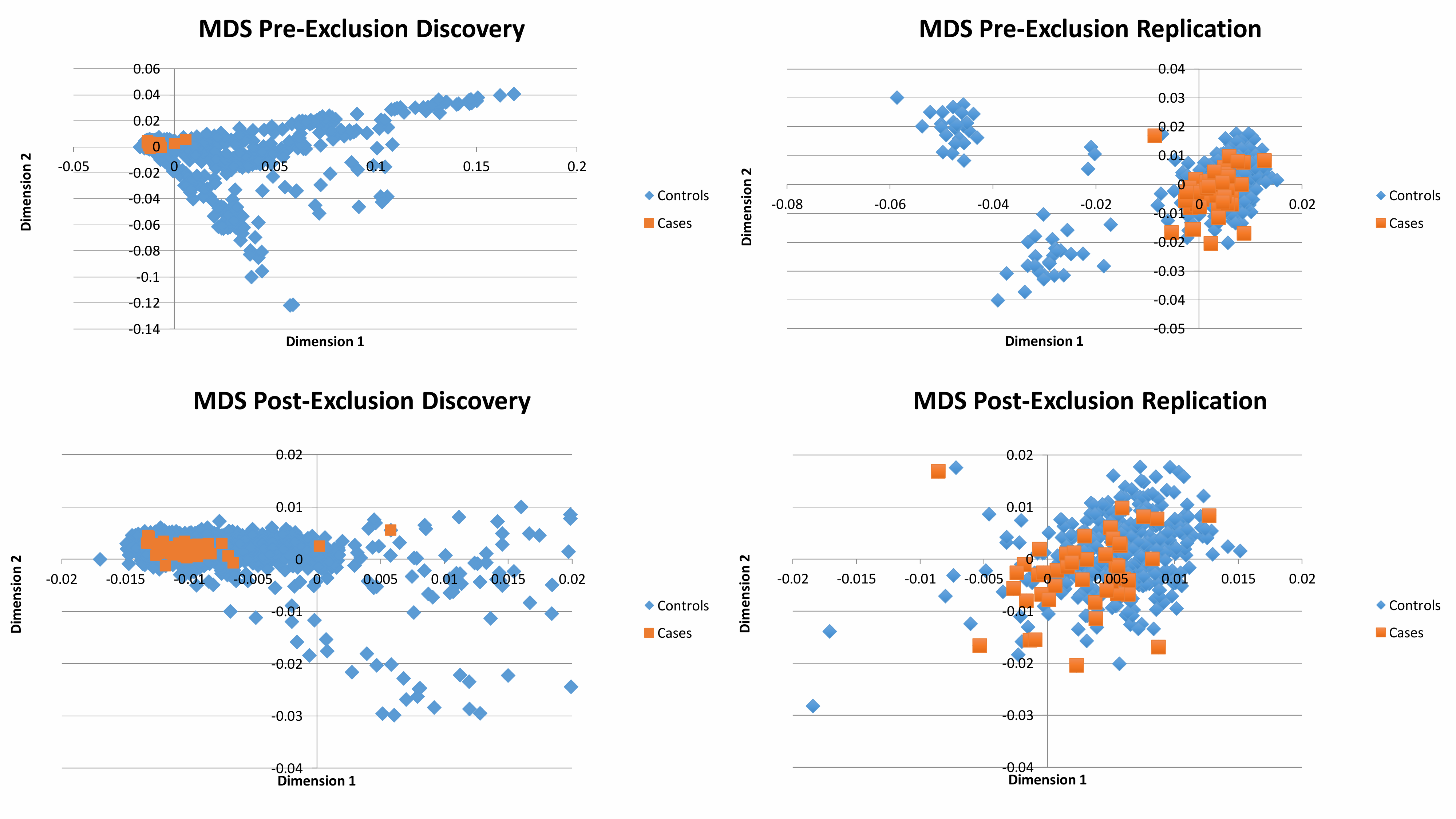

Supplement: Supplementary file 1 [file Image_1.TIF]

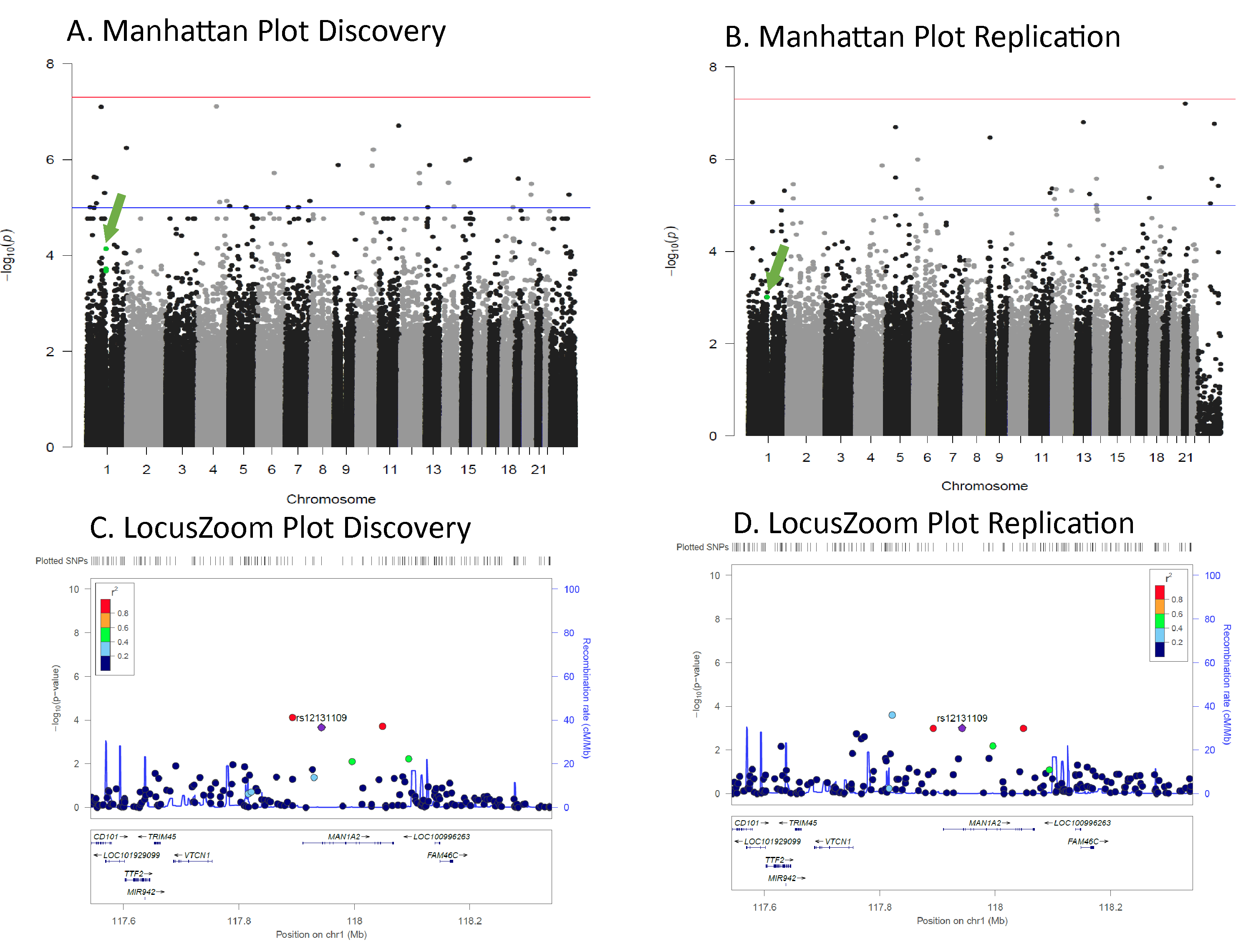

Supplement: Supplementary file 2 [file Image_2.TIF]

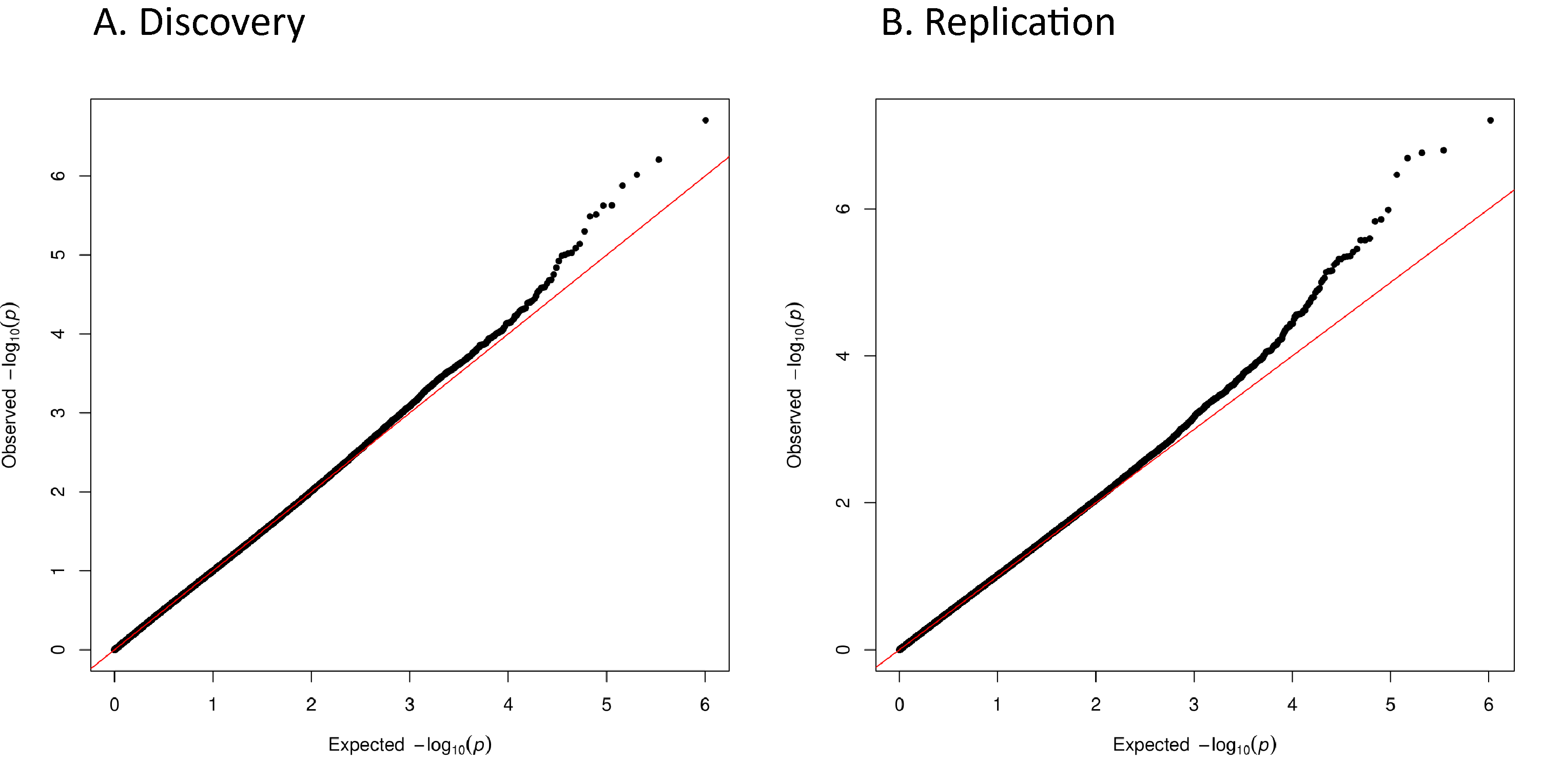

Supplement: Supplementary file 3 [file Image_3.TIF]

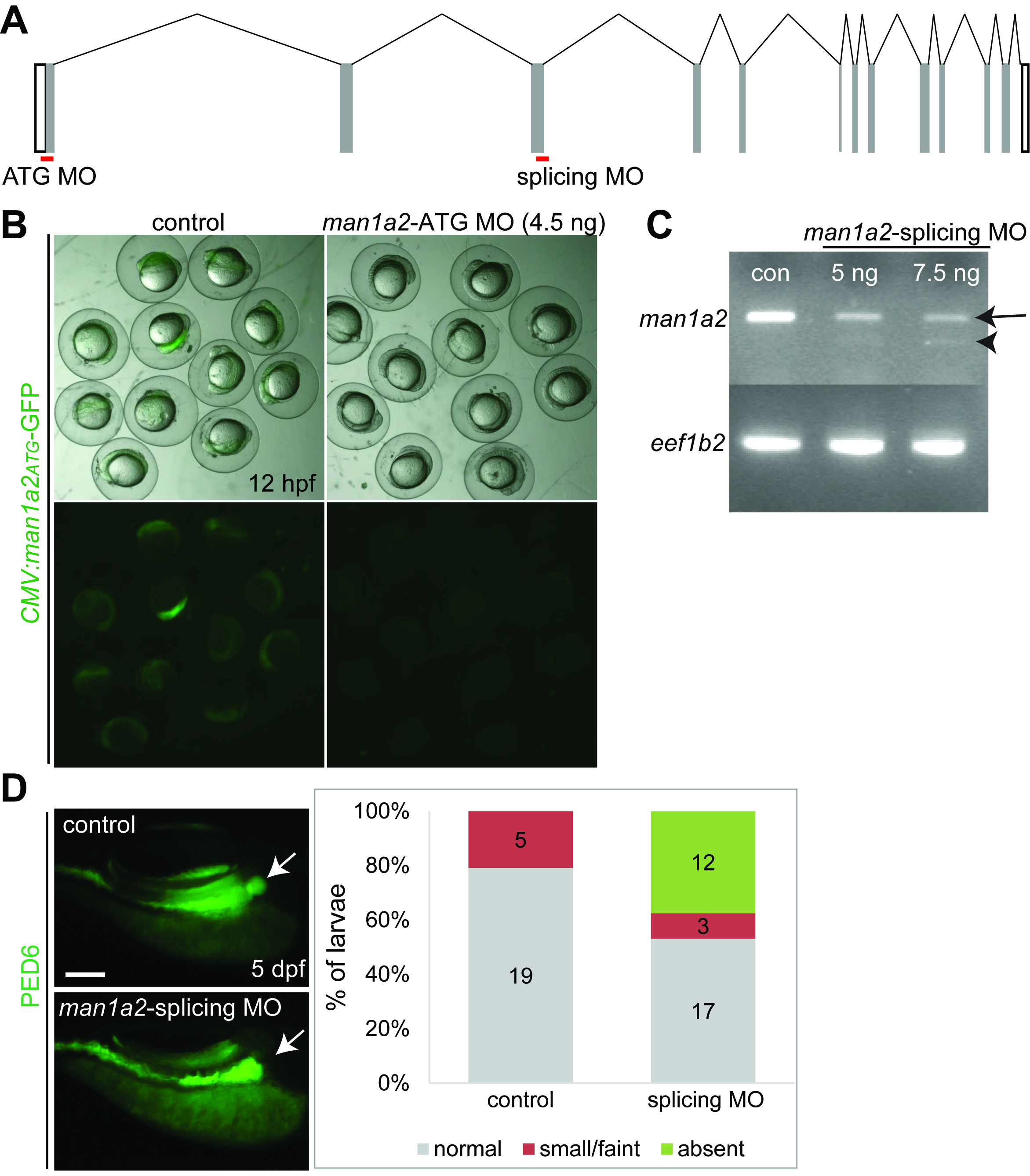

Supplement: Supplementary file 4 [file Image_4.TIF]

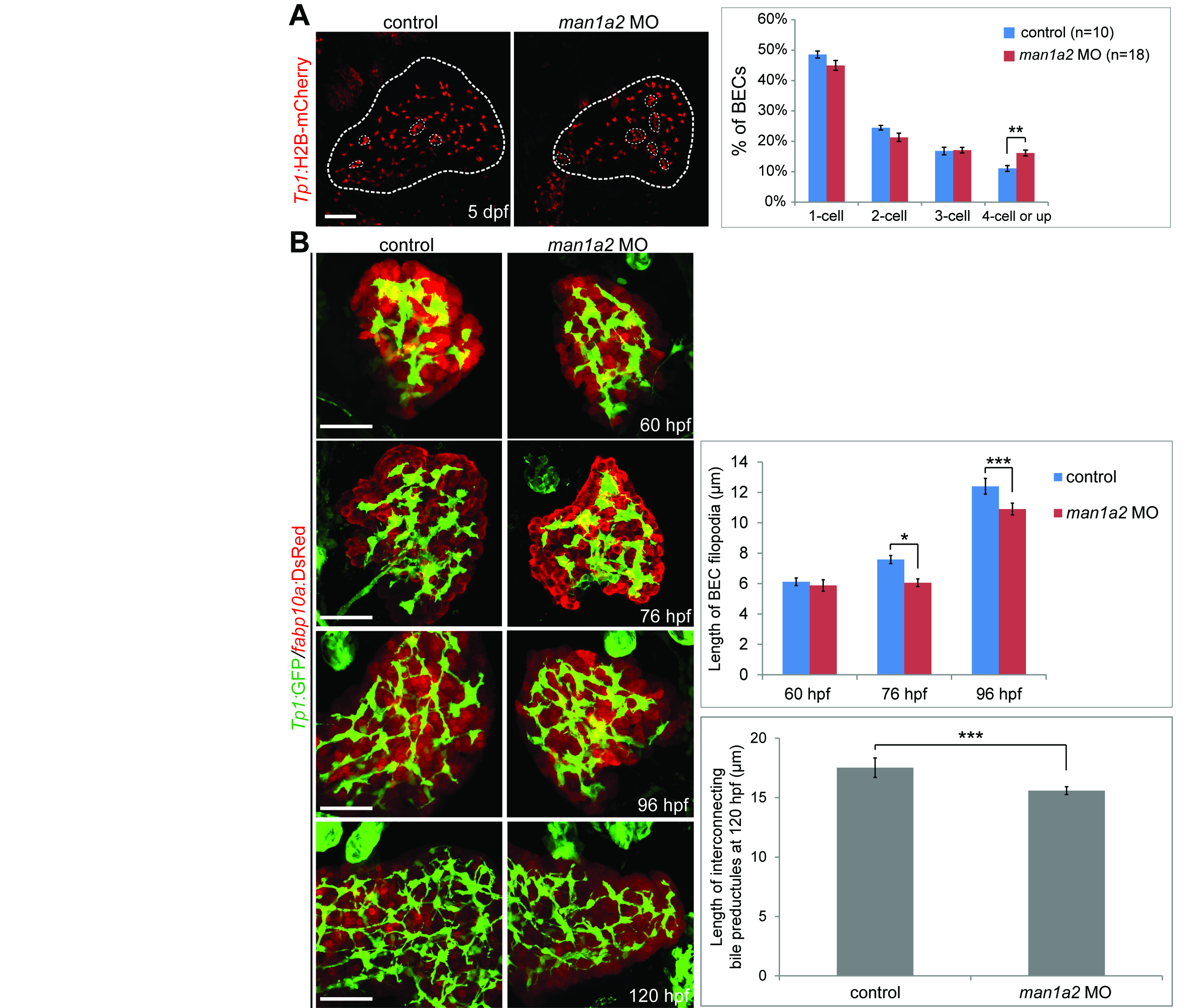

Supplement: Supplementary file 5 [file Image_5.TIF]

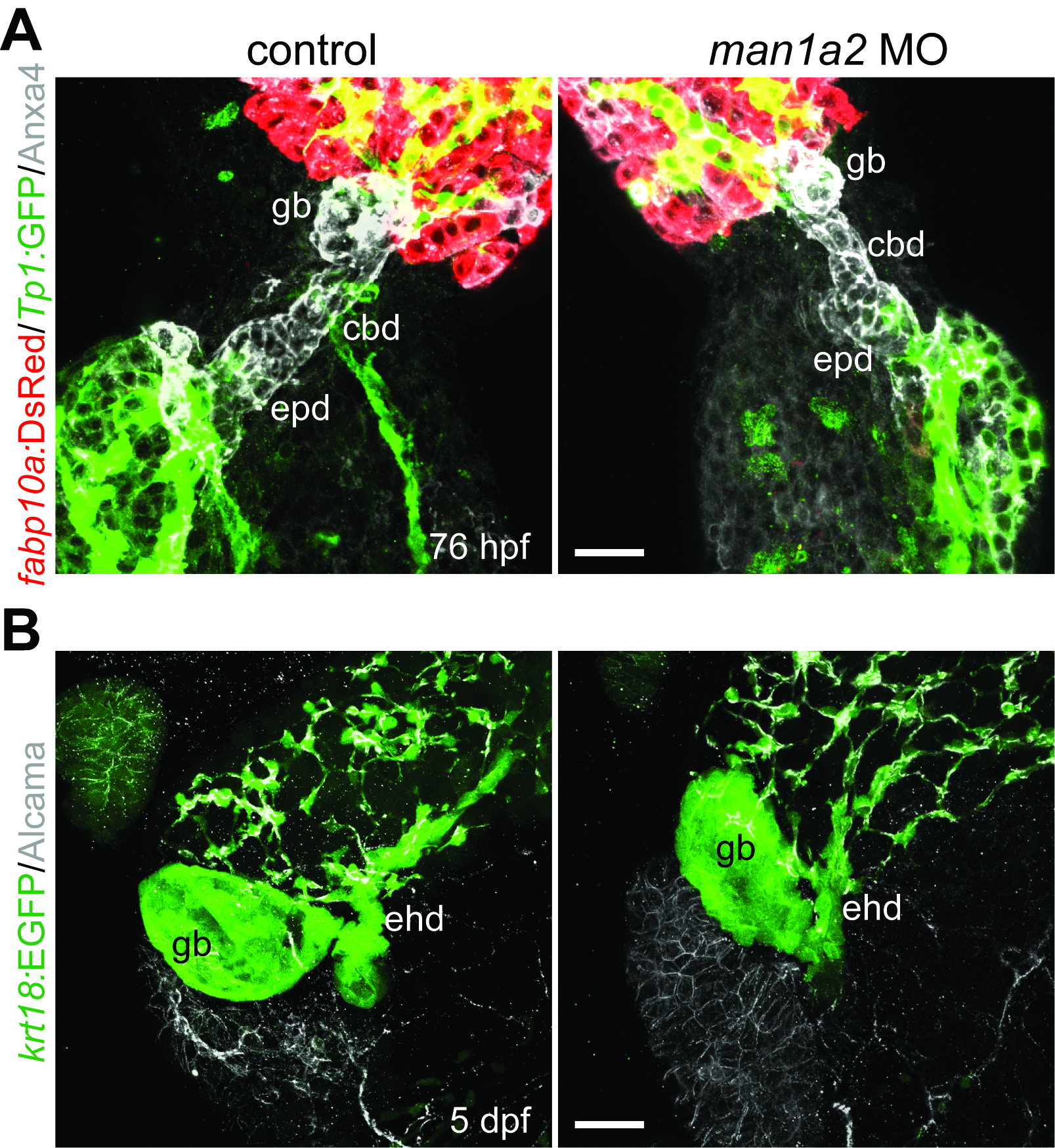

Supplement: Supplementary file 6 [file Image_6.TIF]

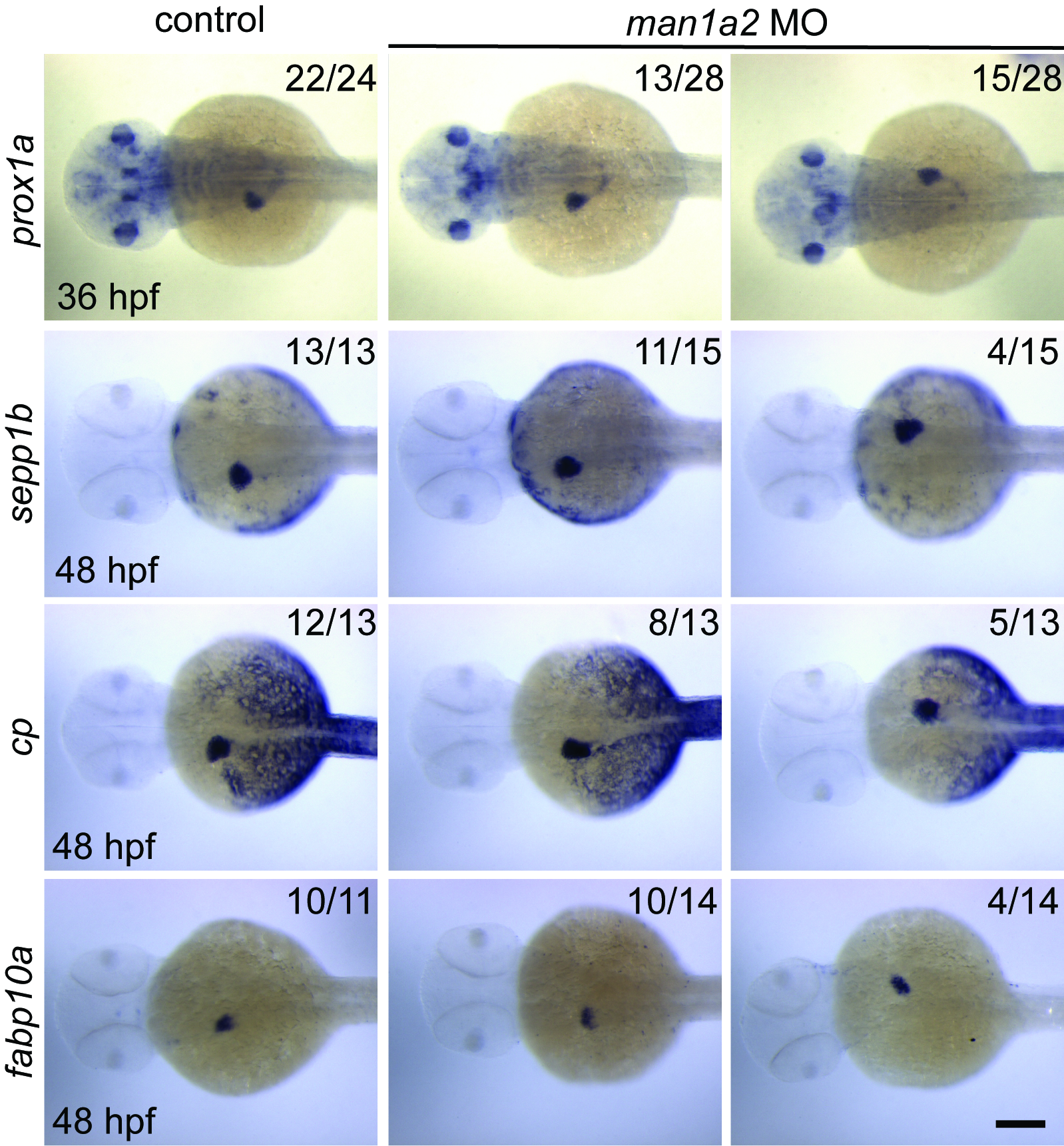

Supplement: Supplementary file 7 [file Image_7.TIF]
