# Supplementary material for: Biliary-Atresia-Associated Mannosidase-1-Alpha-2 Gene Regulates Biliary and Ciliary Morphogenesis and Laterality
Source: Front Physiol. 2020 Oct 30;11:538701. doi: 10.3389/fphys.2020.538701 (PMC7662016; doi:10.3389/fphys.2020.538701)
Supplement: Supplementary file 9 [file Table_2.docx]

Supplement to:

##### The Biliary-Atresia-associated Mannosidase-1-alpha-2 gene regulates biliary and ciliary morphogenesis and laterality.

Juhoon So,1**¶** Mylarappa Ningappa,2**¶** Joseph Glessner,3**¶** Jun Min,4**¶** Chethan Ashokkumar,2 Sarangarajan Ranganathan,5 Brandon W. Higgs,2 Dong Li,3 Qing Sun,2 Lori Schmitt,6 Amy C. Biery,5 Steven Dobrowolski,5 Christine Trautz,2 Leah Fuhrman,2 Molly Christine Schwartz,7 Nikolai Thomas Klena,7 Joseph Fusco,8 Krishna Prasadan,8 Morayooluwa Adenuga ^2^, Nada Mohamed^8^, Qi Yan,9 Wei Chen9, William Horne,^10^ Anil Dhawan,11 Khalid Sharif,^12^ Deirdre Kelly, ^12^ Robert H. Squires,13 George K. Gittes,8 Hakon Hakonarson,3 Victor Morell,14 Cecilia Lo,7 Shankar Subramaniam,4* Donghun Shin,1* Rakesh Sindhi,2*.

1. **Supplementary Methods**
2. **Supplementary References**
3. **Supplementary Figure Legends**
4. **Supplementary Table Legends**
5. **Supplementary Video Legends**

***1. Supplementary Methods:***

**Methods: Human Subjects**. Archived DNA from 137 Caucasian children with BA who received LTx at the Children's Hospital of Pittsburgh was genotyped after informed consent approved by the University of Pittsburgh’s Institutional Review Board (IRB#0405628). These subjects included 112 subjects without and 25 subjects with extrahepatic anomalies. Genome-wide genotypes for healthy Caucasian controls (NC) were provided by the Center for Applied Genomics (CAG) at the Children’s Hospital of Philadelphia in two batches of 1969 and 400 subjects, respectively.

**Genotyping and candidate gene identification.** Fifty-two BA discovery cases and 53 BA replication cases were genotyped respectively with the HumHap Infinium 550K and the Quad Infinium 660k SNP arrays from a single manufacturer (Illumina, San Diego, CA). For comparison, respective controls consisted of 1969 and 400 healthy Caucasian children genotyped with the HumHap Infinium 550K and the Quad Infinium 660k SNP arrays. Thirty-two other Caucasian BA cases were genotyped using TaqMan SNP Genotyping Assays for candidate SNPs rs12131109 (Life Technologies ID; C_31005743_10) and rs7531715 (Life Technologies ID; C_30583409_30).

The Individual-based genotype missingness cut-off for the discovery and replication data was 0.1. The SNP-based genotype missingness cut-off for the discovery and replication data was 0.1. The SNP-based genotype minor allele frequency cut-off for the discovery and replication data was 0.01. The SNP-based genotype Hardy Wienberg Equilibrium p-value cut-off for the discovery and replication data was 0.00005. After excluding one discovery BA case due to missing genotypes above the threshold, 507540SNPs in 44 of 51 discovery BA cases and 1713 of 1969 controls passed QC after excluding 28180 SNPs due to missing genotype data (GENO> 0.10), 14480 SNPs removed based on Hardy-Weinberg equilibrium (HWE) test (p <= 0.00005), and 21538 SNPs which failed minor allele frequency test (MAF < 0.01). The 45 of 53 BA cases and 347 of 400 controls in the replication cohort passed QC at 52219 of 657366 SNPs of after excluding 97795 because of missing genotypes (GENO> 0.10), 12481 removed based on HWE (p <= 0.00005), and 24897 SNPs which failed minor allele frequency test (MAF < 0.01).

Cases and controls with similar genetic substructure were identified with multi-dimensional scaling (MDS) plots and carried forward for allele frequency comparisons using the Cochran-Armitage trend test in PLINK ^1^ and all three SNPs achieved genome-wide significance after adjusting for False Discovery Rate using Benjamini Y method (FDR_BY)^20^.

**Targeted Sequencing.** Targeted sequencing of the *MAN1A2* gene, inclusive of 22.26 kb upstream and 20 kb downstream flanking sequences was performed using Agilent Technologies’ Haloplex target enrichment system and Illumina Hiseq2500 sequencing, and data were analyzed. Briefly, Haloplex target enrichment was performed by the enzymatic shearing of 200 ng of genomic DNA using 8 unique restriction enzyme double digests. The fragmented material was hybridized to biotinylated probes designed against the target region. The hybridization reaction with the Haloplex probes generated single-stranded circular DNA molecules and incorporated a small cassette within each circularized DNA molecule. The cassette contained the barcode/index for the sample, the Illumina P5/P7 sequencing primer sites, and one additional pair of primer binding sites. These latter primer binding sites facilitated the construction of the enriched final library by using the circularized DNA molecules as the template material for PCR. For this Haloplex custom capture design, 18 cycles of PCR were performed to generate the enriched libraries. The amplicons were then purified using AMPure XP magnetic beads and quantified using the Agilent 2100 Bioanalyzer. Molarity readings from the Agilent 2100 Bioanalyzer were used to multiplex/pool the samples prior to paired end sequencing on the Illumina Hiseq 2500 platform. This strategy of amplicon-style target enrichment eliminates the need to optimize and perform multiplex PCR. It also allows for up to 8 unique amplicons to be generated per target region, thus reducing instances of amplicon dropout and providing increased confidence in making SNP calls from target regions that contain sequencing reads from multiple amplicons with unique start and stop points.

The quality and coverage of the sequencing data were checked by FastQC, Integrative Genomics Viewer (IGV) and CalculateHsMetrics from Picard. For data analysis, the reads were first aligned against hg19 human reference genome using Burrows-Wheeler Aligner (BWA).^2^ The aligned reads were then further analyzed with Genome Analysis Toolkit (GATK)^3^ v3.3-1. The average coverage depth of targeted sequencing ranged from 172.13 to 1190.32, with a mean of 642.41. Using GATK, local realignment, variant calling, and variant filtration were done in sequence. The newest HaplotypeCaller function was used for variant calling. Hard filters such as QualByDepth (QD) < 2.0, FisherStrand (FS) > 60, RMSMappingQuality < 40.0, HaplotypeScore > 13.0, MappingQualityRankSumTest < -12.5, and ReadPosRankSumTest < -8 were used to filter out potentially false-positive variants. Additionally, we filtered out the variants 1) with coverage depth less than 20, and 2) with a ratio of reference allele and alternative allele greater than 3.

The extended locus around each BA-associated SNP was then defined by identification of all SNPs in LD at threshold *r*2 ≥ 0.8 using HaploReg v4.1 (4) based on Phase I of the 1000 Genomes project. Variants in this extended locus were explored for impact on gene function with Annovar^5^ and regulatory function (including eQTLs) by HaploReg v4.1^4^ and RegulomeDB,^6^ which both collate data from the Encyclopedia of DNA Elements (ENCODE)^7^

**Human gene expression analysis**. Total RNA was extracted from biopsies of normal liver allografts (NC) before transplantation and explanted BA liver tissue, and the RNA was reverse-transcribed to cDNA using High-Capacity cDNA Reverse Transcription Kit (Thermofisher). Gene expression was measured with qRT-PCR with the 7300 Real Time PCR (Applied Biosystems, Foster City, CA). Target RNA levels were also measured in SYBR Green/ROX qPCR master mix (Fermentas, Glen Burnie, MD) with two pairs of primers for *MAN1A2* gene that cover the exons 6 and 7 region or the exon 10 region (IDT, Coralville, IA) (Supplementary Table 13). Target mRNA levels are expressed relative to GAPDH and fold change gene expression was calculated in BA over normal liver tissue.

**Histology and Immunohistochemistry of human samples.** To validate molecular findings, tissue immunohistochemistry was performed on liver tissue from two normal liver allografts and five children with BA. Four micron thick, paraffin-embedded sections were cut and stained with antibody to MAN1A2 (1:50 dilution, Novus Biologicals, Littleton, CO) along with normal liver as a positive control (8). Controls also included explanted liver tissue from a child each with Alagille’s syndrome and types 1 and 2 progressive familial intrahepatic cholestasis. Each sample was stained on a Ventana Benchmark Ultra automated staining platform, using HIER (heat induced epitope retrieval) with a proprietary buffer from Ventana. Detection was performed using the ultraView DAB detection kit from Ventana and counterstained with hematoxylin.

***Man1a2* siRNA knockdown analysis in reciliating mouse airway epithelial cultures.** For gene knockdown analysis, trachea from C57BL6/J mice were cut lengthwise and placed in Leibovitz-15 medium (Gibco) with 01% Pronase (Sigma) on a shaker at 37°C. After 75 min, fetal bovine serum was added to 10%, the tissue suspension pelleted, then washed twice in stationary culture medium (DMEM/F12 containing 0.02% Ultroser G and antibiotic-antimycotic) followed by vigorous pipetting to disaggregate the tissue pellet. Then the tissue suspension is plated onto rat-tail collagen-coated plates for proliferative growth. After 7 to 9 days when the culture reached confluence, the cells were removed with collagenase treatment (200 IU/ml, Worthington), pelleted, washed with medium and resuspended in suspension culture medium in a T-25 flask on an orbital shaker at 37°C for 7-10 days for reciliation, which is usually observed by day 7-9.

Gene knockdown analysis was conducted in the reciliating tracheal epithelia using previously described methods.^9^ Briefly, confluent tracheal epithelial monolayer cultures were transfected with four *Man1a2* specific siRNA (Qiagen Flexitube siRNA, #1027416) at 80 picomolar in OPTI-MEM (Thermofisher) using Lipofectamine RNAimax (Thermofisher). The transfection cocktail of four siRNA was added to the monolayer tracheal epithelia for 3 hrs at 37°C/5% CO2, followed by processing for suspension culture to promote reciliation as described above. Scrambled siRNA transfection is used as a negative control. The efficacy of siRNA knockdown was confirmed with real time PCR analysis with RNA isolated from the mouse tracheal epithelium 24 hours post transfection. Quantitative real-time PCR was performed using Sybr Green Based-detection (Thermo Fisher Scientific, Waltham, MA) with primers for *Man1a2* gene and housekeeping gene beta actin (IDT, Coralville, IA). All experiments were performed in triplicate. The primers used included: *Man1a2* Forward 1: 5’-acccacgacccaagatacag-3’, *Man1a2* Reverse 1: 5’-ggcacgacttctcaatagcc-3’, *Man1a2* Forward 2: 5’-tatggcgatttacccacgac-3’, *Man1a2* Reverse 2: 5’-ggcacgacttctcaatagcc-3’, *beta-actin* -forward: 5’-ctaaggccaaccgtgaaaag-3’, *beta- actin* reverse: 5’-accagaggcatacagggaca-3’.

The effects of gene knockdown on ciliogenesis and ciliary motion was examined by videomicroscopy using DIC optics under a Leica inverted microscope equipped with a 60X oil immersion lens and a Phantom camera as previously described.^9^ For each knockdown culture, more than ten videos were collected. Using these videos, we measured the percent citation using ImageJ (length of ciliated cell surface divided by entire epithelia surface length) Zebrafish experiments.

**Immunohistochemistry and quantification**: Lung samples were fixed with 4% paraformaldehyde (PFA) in PBS overnight, dehydrated using 30% sucrose overnight then embedded in optimal cutting temperature (OCT) compound, snap-frozen by liquid nitrogen, and sectioned at 7µM. Antigen retrieval was performed (heat and/or acid buffer). Slides were incubated with mouse monoclonal anti-Arl13b antibody (Antibodies Incorporated, Davis, CA) at 4°C overnight and then incubated with Cy3-conjugated secondary antibody (Jackson ImmunoResearch Labs, West Grove, PA) for 1h at RT the following day. Nuclear staining and mounting were performed using Fluroshield with DAPI (Sigma-Aldrich, St.Louis, MO). The percentage of Arl13b+ cells per section were quantified using ImageJ software from at least six sections that were 100µm apart for each mouse.

**Zebrafish strains**. Embryos and adult fish were raised and maintained under standard laboratory conditions. We used the following transgenic lines: *Tg(dusp6:d2EGFP)pt6*,^10^ *Tg(krt18:EGFP)p314*,^11^ *Tg(EPV.Tp1-Mmu.Hbb:EGFP)um14*,^12^ *Tg(EPV.Tp1-Mmu.Hbb:hist2h2l-mCherry)s939*,^13^ and *Tg(fabp10a:DsRed,ela3l:EGFP)gz15*,^14^ [the last three referred to here as *Tg(Tp1:GFP)*, *Tg(Tp1:H2B-mCherry)*, and *Tg(fabp10a:DsRed)*, respectively].

**Morpholino and DNA injections.** *arf6*-ATG MO (5’-GATCTTGGAAAGCATCTTCCCCATG-3’) (8), *man1a2*-ATG MO (5’-CCGGCGTGGTCATATTTTGATGATC-3’), and *man1a2*-splicing MO (5’-AAGAATGTAAACTCACCTCTCTGAT-3’) were purchased from Gene Tools, LLC. Embryos were injected at the one-cell stage with *man1a2*-ATG MO (1.5 or 4.5 ng), *man1a2*-splicing MO (5 or 7.5 ng), or *arf6*-ATG MO (0.5 ng). For validation of the *man1a2*-ATG MO, 20 pg of the plasmid containing the *CMV*:*man1a2ATG*-*GFP* construct, which was generated as previously described,^12^ was injected into the cell at the one-cell stage. To validate the *man1a2*-splicing MO, RT-PCR was performed using a forward (5’-CCGGACAATTCCAAACACAAGC-3’) and a reverse (5’-CAGCCGTATTGTCTGTAGCTG-3’) primer set for *man1a2* and a forward (5’- CCCTCTCAGGCTGATATTGC-3’) and a reverse (5’-TAAGCTGCAAGCCTCTCCTC-3’) primer set for *eef1b2*.

**Whole-mount in situ hybridization and immunostaining.** Whole-mount in situ hybridization was performed as previously described,^15^ using *man1a2*, *myl7*, *foxa3*, *prox1a*, *sepp1b*, *cp*, and *fabp10a* probes. For imaging, stained embryos were mounted in 90% glycerol/PBS. Whole- mount immunostaining was performed as previously described,^16^ using the following antibodies: chicken polyclonal anti-GFP (1:1000; Aves Labs), mouse monoclonal anti-Anxa4 (also named as 2F11; 1:100; Abcam), rabbit polyclonal anti-Abcb11 (1:200; Kamiya Biomedical), mouse monoclonal anti-Alcama (1:20; ZIRC), mouse monoclonal anti-acetylated tubulin (1:200; Sigma-Aldrich), and Alexa Fluor 488-, 568-, and 647-conjugated secondary antibodies (1:500; Life Technologies). For confocal imaging, embryos/larvae were mounted in 1.2% low-melting agarose on a slide glass.

**PED6 and BODIPY C5 assays.** PED6 assay was performed by treating larvae with 0.3 µg/ml PED6 (Life Technologies) for 3 hours as previously described;^17^ BODIPY C5 assay was performed with 0.5 µM BODIPY C5 (Life Technologies) for 2 hours as previously described.^18^ Images for PED6 and BODIPY C5 assays were obtained using epifluorescence and confocal microscopes, respectively. BODIPY C5-treated larvae were briefly rinsed, anesthetized with 0.016% Tricaine/egg water, and then mounted in 1% low-melting agarose for confocal imaging.

**Image acquisition, processing, and statistical analysis.** Zeiss LSM700 confocal and Leica M205 FA epifluorescence microscopes were used to obtain zebrafish image data. Confocal stacks were analyzed using the ZEN 2009 software. The length of BEC filopodia and interconnecting bile preductules and cilia length were measured using the ImageJ software and were shown as means ± SEM (standard error of the mean). The number of BECs and cilia was also counted using the ImageJ software. Unpaired two-tailed Student’s t-test was used for statistical analysis; p<0.05 was considered statistically significant.

**Zebrafish gene expression analysis:** Total RNA was extracted from three different batches of 100 livers of uninjected control and *man1a2* MO-injected larvae at 5 dpf by using RNeasy Mini Kit (Qiagen, Valencia, CA). Whole transcriptome amplification system (WTA2) (Sigma Aldrich, St. Louis, MO) was used to synthesize μg quantities of amplified cDNA starting with ~50 ng of RNA. Quantitative PCR was performed with SYBR Green/ROX qPCR master mix (Fermentas, Glen Burnie, MD) with respective primers (IDT, Coralville, IA) (Supplementary Table 14) and 7300 Real Time PCR (Applied Biosystems, Foster City, CA). Target gene expression was normalized to the house keeping gene, *eef1a1l1*. The relative expression level of genes was shown in fold change calculated using the ΔΔCt method in zebrafish morphants over un-injected controls.

**Evaluating lung and liver development using *Man1a2 +/-* mice***.* All mouse experiments were approved by the Animal Research and Care Committee at the Children’s Hospital of Pittsburgh and the University of Pittsburgh IACUC*. Man1a2 +/-* mice (*Man1a2*^tm1.1Ahe^, #007672., Jackson Labs, Bar harbor, Maine, US) were bred to evaluate whether newborn *Man1A2 -/-* null mice demonstrated abnormal liver architecture in addition to the known lethal changes in the lung,^19^ compared with wild type. Within 30-60 minutes of birth, newborn mice were decapitated, dissected through a midline incision extending from the pubic symphysis cranially into the thoracic cavity, and lung and liver tissue harvested for histological examination and RNA extraction after gross examination for abdominal or thoracic situs inversus. Sections were stained with hematoxylin-eosin, and antibodies to EPCAM and examined by a single pathologist (SR). Total RNA was extracted from livers of mouse pups at first day of life using RNeasy Mini Kit (Qiagen, Valencia, CA) and reverse-transcribed to cDNA using High-Capacity cDNA Reverse Transcription Kit (Thermo Fisher Scientific, Waltham, MA). Reverse transcription quantitative PCR (RTqPCR) was performed with SYBR Green/ROX qPCR master mix (Fermentas, Glen Burnie, MD) with respective with primers for *Man1a2* gene spanning Exon 2, Exon 5-6, and Exon 11-12, and housekeeping gene beta actin (IDT, Coralville, IA) (Supplementary Table 15).

**Evaluating liver histology in *Dnah-/- mice*:** The mutant line b2b1775Clo was identified in a large-scale, recessive, forward genetic, ENU mutagenesis screen elucidating the genetic basis of congenital heart disease.^9^ b2b1775Clo was detected to have complex congenital heart disease associated with heterotaxy through fetal echocardiography. Diagnosis was confirmed through necropsy analysis, episcopic confocal microscopy (ECM), and micro-computed tomography (CT)/micro-magnetic resonance imaging (MRI). Whole exome sequencing revealed a splicing mutation in intron 41 in the primary ciliary dyskinesia (PCD) and Kartagener syndrome associated gene *Dnah11*. Homozygosity of the *Dnah11*^c.6489+2T^ mutation was validated by Sanger sequencing of all mutants and confirmed by breeding multiple generations. *Dnah11* transcript analysis revealed a frameshift mutation resulting in premature termination at the lysine residue at amino acid position 2163. *Dnah+/+* and *Dnah-/-* mice were sacrificed at P0.

**Whole transcriptome RNA sequencing of human BA.** Total RNA sequencing protocol was performed as follows on four human BA recipient liver and three normal liver allografts. Total RNA sequencing protocol: Total RNA libraries were generated using Illumina TruSeq Stranded Total RNA Sample Preparation Guide Rev. E. First cytoplasmic and mitochondrial ribosomal RNA was removed with biotinylated, target-specific oligos and Ribo-Zero rRNA removal beads. The purified RNA was fragmented using divalent cations under elevated temperature, and fragments were copied into first strand cDNA using reverse transcriptase and random primers, followed by second strand cDNA synthesis using DNA Polymerase I and RNase H. After adding a single 'A' base, cDNA was ligated to the adapter, purified and enriched with PCR to create the final cDNA library. Libraries were validated using KAPA Biosystems primer premix kit with Illumina-compatible DNA primers and Qubit 2.0 fluorometer. Quality is examined using Agilent Bioanalyzer Tapestation 2200, and libraries pooled at a final concentration 1.8pM. Cluster generation and 100 bp paired read dual-indexed sequencing was performed on NextSeq 500.

**Whole transcriptome RNA zebrafish man1a2 knockdown morphants.** Total RNA sequencing protocol was performed on pooled liver tissue from all three batches, one from controls and one from *man1a2* morphant zebrafish. Each pool consisted of 100 livers from three batches of zebrafish larvae at 5dpf. The pooled RNA was used to generate mRNA sequencing libraries using Illumina TruSeq Stranded mRNA sample preparation kit. Poly-A containing mRNA molecules were purified using poly-T oligo attached magnetic beads, mRNA fragmented into small pieces using divalent cations, and copied into first strand cDNA using reverse transcriptase and random primers. Strand specificity was achieved by using dUTP in the Second Strand Marking Mix, followed by second strand cDNA synthesis using DNA Polymerase I and RNase H. These cDNA fragments were ligated to single 'A' base and adapter, then purified and enriched with PCR to create the final cDNA libraries which were validated using KAPA Biosystems primer premix kit with Illumina-compatible DNA primers and Qubit 2.0 fluorometer. Quality was examined using Agilent Tapestation 2200.

**Whole transcriptome RNA sequencing of *Man1a2-/-* mouse liver and lung**. Total RNA from liver and lung from three P0 mice each from *Man1a2+/+* (WT) *and Man1a2-/-* (Null mice) was used to generate mRNA sequencing libraries using Illumina TruSeq Stranded mRNA sample preparation kit. Poly-A containing mRNA molecules were purified using poly-T oligo attached magnetic beads, mRNA fragmented into small pieces using divalent cations, and copied into first strand cDNA using reverse transcriptase and random primers. Strand specificity was achieved by using dUTP in the Second Strand Marking Mix, followed by second strand cDNA synthesis using DNA Polymerase I and RNase H. These cDNA fragments were ligated to single 'A' base and adapter, then purified and enriched with PCR to create the final cDNA libraries which were validated using KAPA Biosystems primer premix kit with Illumina-compatible DNA primers and Qubit 2.0 fluorometer. Quality was examined using Agilent Tapestation 2200.The cDNA libraries were pooled at a final concentration 1.8pM. Cluster generation and 75 bp Paired-read dual-indexed sequencing was performed on Illumina NextSeq 500.

**Whole transcriptome RNA sequencing data analysis.**

The widely used R-package, DEseq, was used for the differential expression analysis. The cutoff is alpha=0.05 for unadjusted p-values. The raw p-values were adjusted for multiple testing using Benjamini-Hochberg method^20^ to calculate the false discovery rate (FDR). Genes were selected based on cutoff of 1.2-fold or greater and adjusted p-values <0.05.

***2. Supplementary References:***

1. Purcell S, Neale B, Todd-Brown K, Thomas L, Ferreira MA, Bender D, Maller J, Sklar P, de Bakker PI, Daly MJ, Sham PC. [PLINK: a tool set for whole-genome association and population-based linkage analyses.](http://www.ncbi.nlm.nih.gov/pubmed/17701901) Am J Hum Genet. 2007;81:559-575.
2. Li H, Durbin R. Fast and accurate short read alignment with Burrows-Wheeler transform. Bioinformatics 2009;25:1754-1760.
3. DePristo MA, Banks E, Poplin R, et al. A framework for variation discovery and genotyping using next-generation DNA sequencing data. Nat Genet 2011;43:491-498.
4. Ward LD, Kellis M. HaploReg: a resource for exploring chromatin states, conservation, and regulatory motif alterations within sets of genetically linked variants. Nucleic Acids Res 2012:40:D930-934.
5. Wang K, Li M, Hakonarson H. ANNOVAR: functional annotation of genetic variants from high-throughput sequencing data. Nucleic Acids Res 2010;38:e164.
6. Boyle AP, Hong EL, Hariharan M, et al. Annotation of functional variation in personal genomes using RegulomeDB. Genome Res 2012;22:1790-1797.
7. The ENCODE Project Consortium. An integrated encyclopedia of DNA elements in the human genome. Nature 2012;489:57-74.
8. Ningappa M, So J, Glessner J, et al. The role of ARF6 in biliary atresia. PLoS One 2015;10:e0138381.
9. Li Y, Klena N, Gabriel G, et al. Global genetic analysis in mice unveils central role for cilia in congenital heart disease. Nature 2015;521:520-524.
10. Molina GA, Watkins SC, and Tsang M. Generation of FGF reporter transgenic zebrafish and their utility in chemical screens. BMC Dev Biol 2007;7:62.
11. Wilkins BJ, Gong W, Pack M. A novel keratin18 promoter that drives reporter gene expression in the intrahepatic and extrahepatic biliary system allows isolation of cell- type specific transcripts from zebrafish liver. Gene Expr Patterns 2014;14:62-68.
12. Parsons MJ, Pisharath H, Yusuff S, et al. Notch-responsive cells initiate the secondary transition in larval zebrafish pancreas. Mech Dev 2009;126:898-912.
13. Ninov N, Borius M, Stainier DYR. Different levels of Notch signaling regulate quiescence, renewal and differentiation in pancreatic endocrine progenitors. Development 2012;139:1557-1567.
14. Korzh S, Pan X, Garcia-Lecea M, et al. Requirement of vasculogenesis and blood circulation in late stages of liver growth in zebrafish. BMC Dev Biol 2008;8:84.
15. Alexander J, Stainier DY, Yelon D. Screening mosaic F1 females for mutations affecting zebrafish heart induction and patterning. Dev Genet 1998;22:288-299.
16. Dong PD, Munsno C, Norton W, et al. Fgf10 regulates hepatopancreatic ductal system patterning and differentiation. Nat Genet 2007;39:397-402.
17. Farber SA, Pack M, Ho SY, et al. Genetic analysis of digestive physiology using fluorescent phospholipid reporters. Science 2001;292:1385-1388.
18. Carten JD, Bradford MK, Farber SA. Visualizing digestive organ morphology and function using differential fatty acid metabolism in live zebrafish. Dev Biol 2011;360:276-285.
19. Tremblay LO, Nagy Kovács E, Daniels E. [Respiratory distress and neonatal lethality in](https://www.ncbi.nlm.nih.gov/pubmed/17121831) [mice lacking Golgi alpha1,2-mannosidase IB involved in N-glycan maturation.](https://www.ncbi.nlm.nih.gov/pubmed/17121831) J Biol Chem 2007;82:2558-2566.

20. Benjamini Y, Hochberg Y. Controlling the false discovery rate: a practical and powerful approach to multiple testing. J R Stat Soc Ser B Methodol. 1995; 57:289–300.

***3. Supplementary Figure Legends:***

Supplementary Figure 1. Selecting cases and controls with similar genetic substructure. Lower left-hand box in multidimensional scaling plots shows that 44 of 51 BA cases (red circles) cluster with 1713 of 1969 controls (grey-blue circles) in the discovery cohort (A) and 45 of 53 cases and 347 of 400 controls cluster with each other in the replication cohort (B).

Supplementary Figure 2. Manhattan plots shows genome wide significant SNPs (arrow and circle) rs6657965, rs12131109 and rs7531715 in the MAN1A2 gene in the discovery cohort (A) and replication cohort (B). The blue and red lines on plots represent the suggestive and genome-wide significant association cut-offs respectively. Regional LocusZoom plots (http://locuszoom.org/) show significant SNPs rs6657965, rs12131109 and rs7531715 in the MAN1A2 gene in the discovery cohort (C) and replication cohort (D).

Supplementary Figure 3: QQ-plot of the distribution of observed P-values vs expected P-values in both discovery cohort (A) and replication cohort (B).

Supplementary Figure 4. Validation of *man1a2* MOs. (A) Exon and intron structure of the *man1a2* gene. Red lines underline the target regions of two *man1a2* MOs (ATG and splicing MOs) used in this study. (B) *CMV:GFP* constructs containing the target sequence of the *man1a2*-ATG MO in front of the GFP start codon were injected alone or together with the MO. GFP expression was barely detected in the co-injected embryos. (C) Validation of the *man1a2*-splicing MO by RT-PCR analyses. The arrow points to normal *man1a2* PCR products. The arrowhead points to aberrant PCR products, which were only detected in the splicing MO-injected embryos. *eef1b2* was used as RT-PCR controls. (D) Epifluorescence images showing PED6 accumulation in the gallbladder (arrows). Based on PED6 levels in the gallbladder, larvae were divided into three groups: normal, small/faint, and absent. Graph showing the percentage of larvae in each group. Scale bar: 100 µm.

Supplementary Figure 5. *man1a2* regulates intrahepatic biliary morphogenesis. (A) Confocal images of the liver showing the location of BEC nuclei in the entire liver. The *Tg(Tp1:H2B-mCherry)* line was used to reveal BEC nuclei. Dashed lines outline the liver; dotted lines outline clusters with four or more BECs. Graph showing the percentage of BECs present as a single cells, doublets, triplets, or in clusters of four or more cells. (B) Confocal images of the developing liver showing *Tp1*: GFP (green, BECs) and *fabp10a*:DsRed (red, hepatocytes) expression. The length of BEC filopodia at 60, 76 and 96 hpf and the length of interconnecting bile preductules at 120 hpf were quantified and compared between controls and *man1a2* MO-injected larvae, as shown in graphs. Scale bars: 50 µm. Error bars: ± SEM. ***p<0.001; ****p<0.005; *****p<0.01.

Supplementary Figure 6. The formation of the hepatopancreatic ductal system appears normal in *man1a2* MO-injected larvae. (A) Confocal images showing the hepatopancreatic ductal system at 76 hpf, as revealed by Anxa4 expression. *Tg(Tp1:GFP);Tg(fabp10a:DsRed)* larvae were processed for whole-mount immunostaining with Anxa4 (gray), GFP (green), and DsRed (red) antibodies. (B) Confocal images showing Alcama (gray) and *krt18*: EGFP (green) expression in the gallbladder (gb), the extrahepatic duct (ehd), and intrahepatic ducts at 5 dpf. gb, gallbladder; cbd, common bile duct; epd, extrapancreatic duct; ehd, extrahepatic duct. Ventral views, anterior up. Scale bars: 25 µm.

Supplementary Figure 7. *man1a2* knockdown randomizes liver position. Whole-mount in situ hybridization images showing the hepatic expression of *prox1a* at 36 hpf and of *sepp1b*, *cp*, and *fabp10a* at 48 hpf. *prox1a* is a marker of hepatoblasts/hepatocytes; *sepp1b*, *cp*, and *fabp10a* are hepatocyte markers. Liver position was reversed in 40% of *man1a2* MO-injected embryos. Dorsal views, anterior left. Scale bar: 100 µm.

***4. Supplementary Table Legends:***

Supplementary Table 1. Top-ranked significant SNPs in the discovery cohort. SNPs which achieved p-value ≤ 0.005 and are associated with BA in the discovery cohort of 44 BA cases. For comparison, 1713 controls were used for minor allele frequency comparison.

Supplementary Table 2. Top-ranked significant SNPs in the replication cohort. SNPs from discovery cohort which achieved p-value ≤ 0.005 and are associated with BA in the replication cohort of 45 BA cases. For comparison, 347 controls were used for minor allele frequency comparison.

Supplementary Table 3. Phenotype and genotype at the *MAN1A2* SNPs rs12131109 and rs7531715 in 23 children with extrahepatic anomalies.

Supplementary Table 4. SNPs identified by targeted sequencing. List of 17 intronic SNP, one 3’ UTR SNP and one downstream SNP in the *MAN1A2* gene locus, which are in LD (r2>=0.8) with rs7531715 and rs12131109. These SNPs were among 498 SNPs identified with targeted sequencing of DNA from 43 of 136 children with BA. The 498 SNPs include the two BA-associated SNPs rs7531715 and rs12131109 (yellow highlight). The SNP, rs1092336 (orange highlight) is strongly associated with *MAN1A2* expression in the liver (p=3.42E-19, Schadt et al, 2008).

Supplementary Table 5. *MAN1A2* expression in diseased liver tissue. RT-qPCR analysis of human genes in explanted liver tissue from children with BA and normal controls.

Supplementary Table 6. MAN1A2 protein expression in disease liver tissue. Staining characteristics of normal (n=2) and BA (n=5) liver tissue. BA liver tissue was obtained from five of 121 BA cases.

Supplementary Table 7. RT-qPCR analysis of genes in zebrafish liver tissue.

Supplementary Table 8. Exon level transcript expression in *Man1a2* mice. Relative expression of exon 2, exon 5-6, and exon 11-12 in liver and lung from WT (*Man1a2* +/+) and null mice (*Man1a2* -/-) is shown.

Supplementary Table 9. Differentially expressed genes in the liver whole transcriptome of human BA compared to normal human allograft liver.

Supplementary Table 10. Differentially expressed genes in the liver whole transcriptome of null mice (*Man1a2 -/-*) compared to wild type (*Man1a2 +/+*).

Supplementary Table 11. Differentially expressed genes in the lung whole transcriptome of null mice (*Man1a2 -/-*) compared to wild type (*Man1a2 +/+*).

Supplementary Table 12. Differentially expressed genes in the liver whole transcriptome of *man1a2* knockdown morphants compared to uninjected zebrafish larvae (controls) at 5dpf.

Supplementary Table 13. RT-qPCR primer sequences for human gene expression studies in liver tissues from BA children and normal controls.

Supplementary Table 14. Quantitative RT-PCR primer sequences for gene expression studies in zebrafish liver tissue.

Supplementary Table 15. Quantitative RT-PCR primer sequences for gene expression studies in liver tissue and lung tissue in mouse.

1. ***Supplementary Video Legends:***

Supplementary Video 1. Cilia motility in reciliating mouse respiratory epithelia.

Supplementary Video 2. Cilia motility in reciliating mouse respiratory epithelia after si-RNA-mediated *Man1a2* knockdown.
